# Factors Affecting the Formation and Treatment of Thrombosis by Natural and Synthetic Compounds

**DOI:** 10.3390/ijms21217975

**Published:** 2020-10-27

**Authors:** Anna Lichota, Eligia M. Szewczyk, Krzysztof Gwozdzinski

**Affiliations:** 1 Department of Pharmaceutical Microbiology and Microbiological Diagnostic, Faculty of Pharmacy, Medical University of Lodz, 90-235 Lodz, Poland; anna.lichota@umed.lodz.pl (A.L.); eligia.szewczyk@umed.lodz.pl (E.M.S.); 2 Department of Molecular Biophysics, Faculty of Biology and Environmental Protection, University of Lodz, 90-236 Lodz, Poland

**Keywords:** thrombosis, hypoxia, oxidative stress, saponins, flavonoids, anticoagulants

## Abstract

Venous thromboembolism (VTE) refers to deep vein thrombosis (DVT), whose consequence may be a pulmonary embolism (PE). Thrombosis is associated with significant morbidity and mortality and is the third most common cardiovascular disease after myocardial infarction and stroke. DVT is associated with the formation of a blood clot in a deep vein in the body. Thrombosis promotes slowed blood flow, hypoxia, cell activation, and the associated release of many active substances involved in blood clot formation. All thrombi which adhere to endothelium consist of fibrin, platelets, and trapped red and white blood cells. In this review, we summarise the impact of various factors affecting haemostatic disorders leading to blood clot formation. The paper discusses the causes of thrombosis, the mechanism of blood clot formation, and factors such as hypoxia, the involvement of endothelial cells (ECs), and the activation of platelets and neutrophils along with the effects of bacteria and reactive oxygen species (ROS). Mechanisms related to the action of anticoagulants affecting coagulation factors including antiplatelet drugs have also been discussed. However, many aspects related to the pathogenesis of thrombosis still need to be clarified. A review of the drugs used to treat and prevent thrombosis and natural anticoagulants that occur in the plant world and are traditionally used in Far Eastern medicine has also been carried out.

## 1. Introduction

Thrombosis can occur in either veins or arteries. In the veins, it leads to DVT and a pulmonary embolism, referred to as VTE. In turn, in the arteries, it most often causes myocardial infarction, ischemic stroke, and acute limb ischemia [1]. The term DVT usually refers to a blood clot that forms in a deep vein, most often in the large veins that run through the calf muscles and eventually leads to pain and swelling in the leg. If the thrombosis is not treated, a pulmonary embolism may occur [2].

Venous thrombosis is becoming more common and is associated with our lifestyle. It can occur very quickly within an hour or over a long period with worsening symptoms. Long hours of travel, e.g., by plane or car, contribute to thrombosis. Moreover, hormonal therapy, including contraception, is also a contributing factor. Other factors conducive to the development of thrombosis can also include surgery, immobilization as a consequence of a procedure or caused by a long-term treatment, delivery, infectious diseases, pre-existing vein diseases, excessive coagulation, high haematocrit (increase blood viscosity), the use of drugs that irritate the endothelium, and varicose veins [3].

Thrombosis may also occur in pregnant and postpartum women. It affects not only people sitting motionless but may occur after intensive effort as well, and it can also occur after dehydration of the body. It also applies to patients undergoing an orthopaedic surgery. Diabetes, hypertension, but also smoking and being overweight, are additional risk factors for thrombosis [4]. 

Other factors favouring thrombosis are the age of the patients, a sedentary or standing lifestyle, lack of physical activity, obesity, as well as existing cardiovascular diseases such as hypercoagulability of the blood and the presence of varicose vein and recent surgery but also rheumatism, diabetes, smoking, and others [5,6]. Cases of thrombosis are also associated with intravenous drug users (IDUs) in whom 22% of DVT cases were reported. It is estimated that the number of drug users in the world ranges from 11 to 21 million including 4–47.6% of people with DVT [7].

DVT is associated with the formation of a blood clot in a deep vein in the body. However, blood clots can occur most often in the large veins of the calf and thigh muscles. Depending on the size, a blood clot that forms in a vein can narrow the vessel or close it completely. The consequence is the appearance of pain and swelling in the leg, as well as of serious complications such as a pulmonary embolism when a blood clot travels to the lungs leading to the inhibition of blood flow [5].

The clots can also break away and be transferred from the flowing blood to the cerebral vessels leading to an ischemic stroke. Therefore, it is very important to quickly diagnose DVT and immediately start its treatment [2]. Essential in the formation of thrombosis are endothelial cells (ECs), which provide many haemostatic regulatory molecules. The normal endothelium has anticoagulant and antithrombotic properties. Damage to the vessel causes adhesion of the platelets with the participation of the von Willebrand factor (vWF) secreted by the cells. ECs also produce tissue plasminogen activator (t-PA), its inhibitor, and an inhibitor of the tissue factor (TF) pathway. The endothelial surface is additionally the site of thrombin inactivation by antithrombin (AT) and conversion of prothrombotic thrombin by thrombomodulin into anticoagulant protein. In addition, thrombomodulin cooperates with protein C, and the thrombin-activated fibrinolysis inhibitor maintains the endothelial microenvironment in an anti-inflammatory and anticoagulant state [8,9]. Additionally, platelet activation performs a pivotal role in both infection-induced thrombosis and the immune response against pathogens.

Common symptoms of venous thrombosis include swelling, red and tense skin, sometimes a bluish tinge, and an increase in temperature in the extremity. Other symptoms are pain in the foot, calf, and lower knee, which disappears when the limb is lifted. However, one of the dangers of this serious disease is that in many cases it develops asymptomatically, and the patient is unaware of thrombosis. This is the case when the clot is attached to the wall of the vessel and does not completely close the vessel lumen, and some of the flowing blood is taken over by the surrounding smaller vessels. In addition, the blood clot may shrink, which results in an increased blood flow. In the best situation, the blood clot dissolves, and the vessel is restored. Unfortunately, endogenous protective and repair mechanisms are not always sufficient, and the formation of blood clots often leads to complications.

This review describes the causes of vein thrombosis and the basic mechanism of clot formation regarding the intrinsic and extrinsic pathways. Then, we discuss the influence of platelets, endothelial cells, and microorganisms on the development of thrombosis. In the next chapter, we show the role of hypoxic conditions in endothelial dysfunction (ED) by different factors including ROS. The paper reviews available drugs commonly used to fight venous thrombosis. We discuss various treatment strategies used in thrombosis including FX inhibitors, thrombin, antiplatelet therapy (GP IIb/IIIa, GP VI, GP Ib, and other inhibitors), and emerging drugs such as PAR-1 and PAR-4 antagonists and protein disulphide-isomerase (PDI) and phosphatidylinositol 3 kinase β (PI3Kβ) inhibitors. In addition, we describe other natural compounds that have been shown to potentially reduce thrombosis. We present natural anticoagulants from the plant world and traditionally used in Far Eastern medicine.

## 2. The Basic Mechanism of Blood Clotting

This section presents blood coagulation pathways and the participation of various coagulation factors in haemostasis. Discussing this topic is important as the rest of the work will present drug-inhibitors of pathways and coagulation factors in thrombosis. Blood clotting is a natural physiological process that prevents blood loss in the case of a damaged vessel. The blood clotting process is one of the body’s defences when the continuity of the vascular bed is interrupted. The basis of the blood coagulation process is the formation of a clot from fibrin with the participation of thrombin [10]. 

**Figure 1:**
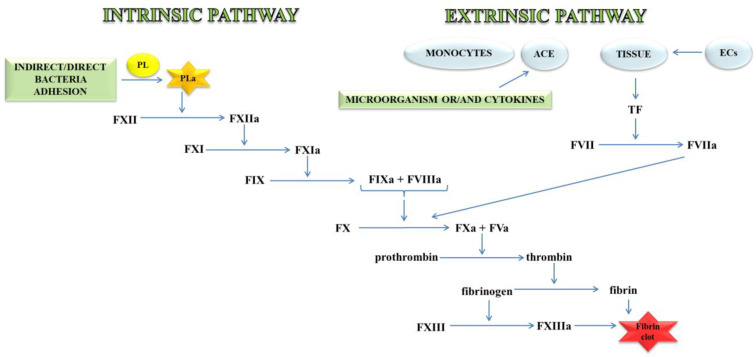
A blood coagulation cascade including the internal and external paths. The former of these pathways is initiated by the contact of FXII with a negatively charged surface or indirectly through the involvement of polyP and blood platelets (PL). In contrast, the external pathway is initiated by FVII activation by tissue factor (TF) on the surface of monocytes, microparticles, activated endothelial cells (ECs), or cells in the damaged vessel wall, which exhibit TF. Both pathways lead to the activation of FX factor (prothrombinase), which converts inactive prothrombin into thrombin. The next stage is the transformation of fibrinogen to fibrin, which forms a fibrin clot; abbreviation: activated platelets (PL-a), angiotensin-converting enzyme (ACE).

Thrombin, an important molecule in the clotting process, cleaves fibrinogen, transforming it into fibrin, which is the main component of the clot. In a further stage, fibrin is cross-linked with the active FXIII to a form resistant to the action of fibrin-degrading enzymes (Figure 1) [11].

The external mechanisms are associated with TF, initiated by tissues or monocytes. TF is an integral type I membrane protein (glycoprotein). A fragment of this glycoprotein is exposed on the membrane surface and binds with very high affinity to FVII. Complex TF-FVII is rapidly converted by limited proteolysis to the activated form of FVII [12]. The internal mechanism occurs due to the action of FXII with the surface having a negative charge. Both mechanisms lead to the activation of the enzymatic transformation cascade FX. The spatial network of a clot is stabilised by transglutaminase, FXIII. In addition, damage to the vessel leads to the discovery of the subendothelial layer, to which the platelets of blood immediately adhere that, as a result of activation, form a clot, inhibiting bleeding from a damaged arteriole, small vein or capillaries. The formation of the clot is caused by the presence of adhesive proteins that participate in the bonding of the tiles between them. The most important protein involved in this process is the platelet integrin αIIbβ3 also called the glycoprotein (GP) IIb/IIIa receptor. In the blood coagulation cascade, other factors such as FVII, FIX, FX, FXI, FXII and activated forms are also involved: FVa, FVIIa, FVIIIa, FIXa, Fxa, FXIa, FXIIa, FXIIIa, and PolyP [13]. Briefly, the process of blood clotting under conditions of haemostasis has been described, taking into consideration the internal and external pathways and coagulation factors.

## 3. Causes of Vein Thrombosis

Already more than 100 years ago, Rudolf Virchow stressed that the cause of thrombosis is the damage to the sinus, endothelial damage, coagulation disorders that lead to an increase in blood viscosity and slow its flow. Arterial thrombosis occurs under conditions of severe shear and is associated with platelets. In contrast, venous thrombosis occurs with low shear flow and mainly around the intact endothelial wall. Arterial thrombosis is associated with the formation of platelet-rich clots in the arteries as a result of atherosclerotic plaque rupture. The detachment of the thrombus can lead to a heart attack, stroke, transient ischaemic attack and limb and intestinal ischemia. On the other hand, thrombosis is related to the formation of clots in the veins of blood cells, low in platelets but rich in fibrin and plasma clotting factors. In thrombosis under hypoxic conditions, a thrombus forms, which causes increased blood clotting activity. Unlike arterial thrombosis, thrombosis causes ischemia at the site of origin. Thrombosis is promoted by the presence of cancer, especially lung and pancreatic cancers, which is associated with disturbances in homeostasis and the clotting process [14].

Blood flow in deep veins is very slow and, hence, the most common thrombosis occurs in these vessels, where the main mechanism forcing blood flow is the spasm of the shins. However, this mechanism stops working when the muscles do not shrink for a long time. Such a phenomenon occurs most often after surgery, in diseases leading to being bedridden, and in people with lower-limb paralysis. The frequency of complications is higher in patients undergoing an orthopaedic surgery than in others; this applies to, for example, a hip fracture. DVT in operated people is initiated by several of factors, such as the release of TF in the operative field, the release and absorption of thrombin from the surgical wound, and imbalance in the fibrinolytic system [15]. Besides, intravenous administration of general anaesthetics leads to a decrease in blood flow. In turn, medications from the curare or succinylcholine group that cause immediate muscle relaxation, reduce the effect of the muscle on the venous vessels and lead to a slowdown in blood flow to a minimum [16]. 

In most patients, sepsis also leads to the activation of the coagulation system and, consequently, thrombosis. Inflammation in the vein wall initiates the formation of a thrombus in a normal vessel. The first stage of clot formation is the activation of ECs, platelets, and leukocytes associated with the induction of inflammation and the release of microparticles that trigger the coagulation system by induction of the TF [17,18]. The thrombus is formed when platelets and leukocytes adhere to the vessel wall, and it is further promoted by inflammation and tissue factor as well as proinflammatory cytokines that activate coagulation. Leukocyte adhesion occurs when P-selectin glycoprotein ligand-1 (PSGL-1) present on the surface binds P-selectin expressed on activated endothelial cells. When P-selectin is also exposed to the surface of blood platelets, it binds to leukocytes leading to inflammation. Furthermore, exposure of P-selectin to the surface of platelets leads to rapid exposure of TF in the blood through monocytes contributing to intravascular coagulation [19].

Thrombosis is not always associated with the occurrence of complications. Occasionally, the thrombus is partially or completely dissolved in the vessel. Sometimes this process may last longer before it is completely dissolved, displaying practically no symptoms. During this period, part of the function related to the blood flow will be taken over by the smaller vessels. DVT has typical signs and symptoms, including pain, swelling, heat, redness or discoloration, and distention of surface veins. However, about half of people with this disease have no symptoms [20]. The presence of a clot can be diagnosed with an X-ray or less invasive methods such as conventional contrast venography, ultrasound, computed tomography (CT), or magnetic resonance imaging (MRI) scans [5].

Thrombosis risk factors are summarized and the mechanism of thrombus formation and the symptoms accompanying this process are described.

### 3.1. Hypoxia and Thrombus Formation

It is believed that hypoxia is the main cause of the development of mechanisms that lead to thrombosis. Hypoxia regulates endothelial pro- and anticoagulant properties. Its function is also to regulate the expression of P-selectin on the endothelium. This process leads to the recruitment of leukocytes or leukocyte microparticles containing TF, which can be an impulse to initiate a thrombotic response [21]. It has recently been shown that hypoxia of the whole body causes venous thrombosis [22,23]. It was reported that 73% of mice developed a thrombus after one-hour stenosis of the inferior vena cava (IVC) [22].

As shown in the mouse models, the size of the thrombus reaches a maximum after 1–2 days, followed by the process of its fragmentation, which depends on the composition, the participation of leukocytes, cytokines, metalloproteinases, and T lymphocytes in it. Clot formation may be related to flow stagnation. The result of stagnation is hypoxia, which develops in the pockets of the venous valves [24]. Such a situation may be caused by a drop in blood pressure and insufficient efficiency of the muscle pump of the calf. In about 50% of patients in whom venous thrombosis occurs, it is impossible to determine the underlying causes. Such cases of VTE are defined as spontaneous or idiopathic. The cause of stagnation in the flow may also be a sitting position during long-haul flights or mountain climbing. In addition, blood stasis is positively correlated with age [25]. Reduced blood flow leads to local hypoxia of the veins. It is known that low oxygen pressure may be harmful to tissues [26].

**Figure 2:**
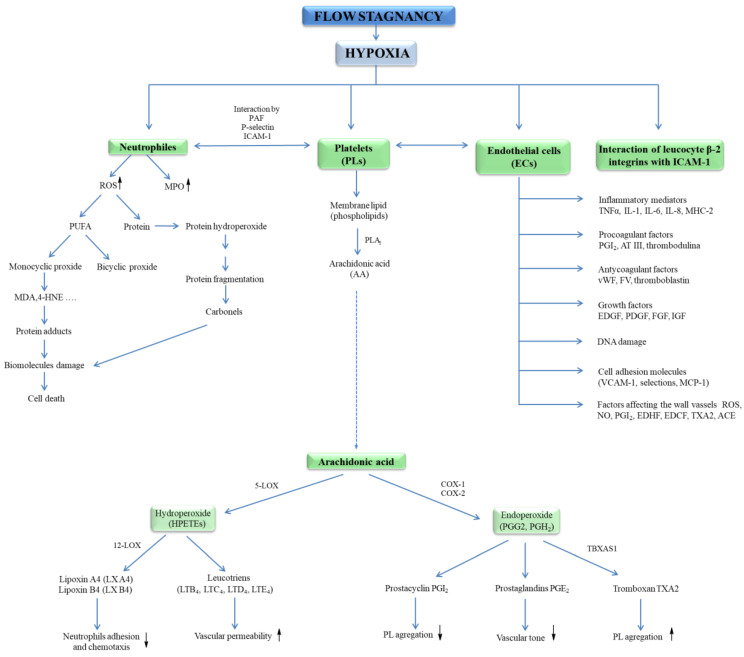
Neutrophils, platelets, and endothelial cells interactions in hypoxia lead to blood cell response. Activation of neutrophils causes a respiratory burst and an increase in myeloperoxidase (MPO) activity. Release of ROS initiates the oxidation of proteins, lipids, and DNA. In contrast, activation of ECs leads to the secretion of procoagulant factors (PGI, AT III, thrombomodulin), growth factors (EDGF, FGF, IGF, PDGF), inflammatory mediators (IL-1, IL-6, IL-8, TNF-α, MHCII), factors regulating vascular wall tension (ROS, NO^•^, PGI2, EDHF, ACE, TXA2, EDCF) as well as release of anticoagulant agents (vWF, FV, thromboplastin), endothelial cell-adhesion molecules (VCAM-1, selectins, MCP-1). In turn, activation of blood platelets leads to the release of PGG2, PGH2, TXA2. Abbreviation: angiotensin-converting enzyme (ACE), antithrombin III – (AT III), deoxyribonucleic acid (DNA), endothelial cells (ECs), endothelium-derived contracting factor (EDCF), endothelium-derived growth factor (EDGF), endothelium-derived hyperpolarizing factor (EDHF), fibroblast growth factor (FGF), insulin-like growth factor (IGF), interleukin (IL), monocyte chemoattractant protein 1 (MCP-1), major histocompatibility complex II (MHCII), myeloperoxidase (MPO), nitric oxide (NO^•^), platelet-derived growth factor (PDGF), prostaglandin G2 (PGG2), prostaglandin H2 (PGH2), prostacycline (PGI), reactive oxygen species (ROS), tumour necrosis factor α (TNF-α), tromboxan A2 (TXA2), vascular cell adhesion molecule (VCAM), von Willebrand factor (vWF).

Hypoxia has been shown to initiate exocytosis and the release of vWF and P-selectin, supporting neutrophil binding [27]. Low oxygen pressure leads to the expression of intercellular adhesion molecule (ICAM-1) and vascular cell adhesion molecule (VCAM-1), but also the expression of angiotensin-converting enzyme (ACE); metalloproteinases; and the activation of leukocytes, mast cells, and ECs in the venous wall, as well as the release of inflammatory mediators (Figure 2) [28]. The increase in the expression of adhesion molecules induced by proinflammatory cytokines such as tumour necrosis factor alfa (TNF-α), interleukin-1 (IL-1), and IL-6, interferon gamma (IFN-γ) leads to the proliferation of smooth muscle cells, which accumulate in the place of the damaged vessel causing an increase in the thickness of its wall. In this condition, recruit leukocytes and platelets lead to thrombosis. Moreover, Gupta et al. [23] showed on the model of venous thrombosis that hypoxia initiates the formation of thromboembolic systems. The same group of researchers reported that a marked, increased expression of inflammasome is a component of the innate immune system (NLRP3), caspase-1, and IL-1β in patients with clinically established venous thrombosis. It has been proved that the activation of this complex is an early response to low oxygen pressure and is regulated by hypoxia-inducible factor-1 alfa (HIF-1α). It was shown that activated macrophages lead to endothelial dysfunction (ED) in hypoxia. Under hypoxic conditions, macrophages release HIF-1α, HIF-2α, which leads to an increase in induced nitric oxide synthase (iNOS), endothelin converting enzyme (ECE-1), and cyclooxygenase 2 (COX-2) and a simultaneous decrease in prostacyclin synthase (SBSI) (Figure 2) [29].

Additionally, hypoxia promotes inflammation. The accumulation of inflammatory cells in many organs, vascular leakage, an increase in the level of serum cytokines, and leukocyte activation have been demonstrated in mice exposed to low oxygen pressure [30,31,32]. The authors also provide evidence that HIF-1 may be involved in bacterial infections. It was shown that bacterial lipopolysaccharide (LPS) initiated the increase in HIF-1α transcription [33]. This problem is of particular importance because ischemia of organ transplants increases the risk of inflammation and failure of the transplanted organs or rejection of the transplant. Hypoxia leads to the migration of HIF-1α and the HIF-1β subunit into the cell nucleus where, after binding to the hormone response element (HRE), they induce transcription of many genes, including nuclear factor kappa B (NF-κB) and toll-like receptor (TLR), and deoxyribonucleic acid (DNA) damage. It was noticed that TLR4 contributes to the inflammation in the transplanted organ and its damage. Affecting the TLR4 signalling may be important in the prevention or treatment of acute renal transplant injury [34,35]. In turn, NF-κB and its family regulate inflammation and affect immune responses and control tissue homeostasis [36].

Hypoxia stimulates the production of ROS, which may initiate the activation of the ECs, leading to the expression of adhesion receptors and the recruitment and extravasation of leukocytes [34,37]. Moreover, the action of ROS leads to the formation of an immune complex, complement and neutrophil activation, and secondary production of oxygen free radicals and other reactive compounds. Activation of the complement system causes its deposition on the wall of the vessel, initiating the inflammation progression [38,39]. Hypoxia leads to oxidative stress in the cells, which is the cause of inhibition of the iron-sulphur (Rieske) protein in complex III of electron transport chain (ETC) by damaged cytochrome. Under this condition, ubiquinol is oxidised to ubisemiquinone, which enhances the oxidative stress [40]. In physiological conditions (normoxia), the cytochrome oxidase present in complex IV of ETC reduces oxygen to water but under hypoxia conditions, it produces nitrous oxide from nitrite [41].

Cytochrome c oxidase of the mitochondrial respiratory chain is one way of inactivating nitric oxide (NO^•^) in cells under hypoxic conditions [42]. Additionally, in hypoxia and inflammation, superoxide (O_2_^•−^) and hydrogen peroxide (H_2_O_2_) can be generated with xanthine oxidase [43]. In hypoxic conditions, NO^•^ synthesis can be catalysed in the blood by haemoglobin (Hb), while in the tissues by xanthine oxidase, neuroglobin, myoglobin, and cytoglobin [44]. At low oxygen pressure, a strong peroxynitrite (ONOO^−^) oxidant can be produced instead of superoxide in the reaction of NO^•^ with O_2_^•−^ [40]. The influence of hypoxia on the release of various active substances leading to the formation of a vascular clot is shown.

### 3.2. Role of Patogens and Oxidtive Stress 

ROS production is the response of the host’s innate immunity to microbial invasion. Oxygen free radicals are an effective weapon against pathogens that prevent tissue colonisation by microorganisms [45]. Generation of ROS can be stimulated by “danger signal molecules” such as extracellular adenosine triphosphate (eATP) released by cells infected or near death. Production of ROS in host cells via the eATP-P2X7 receptor has been shown to be an important modulator for controlling chronic infection and inflammation. This mechanism can explain the persistence of microbes through regulation of ROS signalling under physiological conditions [46].

Bacteria are involved in vascular thrombosis and their strategy is related to the release of various toxins, enzymes and other substances. For example, superantigens (type I toxins) produced by *Staphylococcus aureus* and *Streptococcus pyogenes* damage host cells. Haemolysins and phospholipases (type II toxins) destroy cell membranes. In contrast, type III toxins, also known as A/B toxins, interfere with host cell defence systems to allow dissemination in organs [47]. Streptococcal haemolysins, streptolysins, lead to complete lysis of red blood cells (RBCs). There are two streptolysins: streptolysin O (SLO) and streptolysin S (SLS). SLO exotoxin secreted by group A streptococci and *Streptococcus dysgalactiae* is sensitive to oxygen. Group A streptococci and *Streptococcus dysgalactiae* interact with membrane of red and white blood cells but also macrophages, and platelets. SLS, in turn, is also released by group A streptococci, but is resistant to oxygen. SLS also affects immune cells, including polymorphonuclear leukocytes and lymphocytes. In addition, SLS is thought to prevent the regeneration of the host’s immune system [48]. Shigatoxigenic *Escherichia coli* (STEC) produces Shiga toxin, but the pathogenic mechanism of this bacterium is also associated with the release of enterohaemolysin. This toxin causes cell lysis by a mechanism similar to that of a detergent action [49,50]. 

In turn, bacterium *Staphylococcus aureus* produces a cytolytic toxin that leads to the release of oxyhaemoglobin (oxyHb). The breakdown of oxyHb leads to the secretion of haem, which bacteria use as a source of iron for their growth. In the host organism both haemoglobin molecule and released haem exhibit bactericidal activity by catalysing the reaction with the production of ROS. Additionally, oxyhaemoglobin as a result of autoxidation turns into methaemoglobin (metHb) with superoxide generation. In turn, MetHb exhibits pseudoperoxidase activity capable of catalysing the production of O_2_^•-^, which is a precursor to other ROS [51,52,53].

On the other hand, release of ROS is bactericidal, but it is also associated with damage to the host’s tissues. However, the presence of glycoproteins such as haptoglobin (Hp), hemopexin (Hx) and apoplipoprotein A-I (apoAI) binding oxyHb inhibits oxidative stress [53]. Haemolysis, as a consequence of bacterial infection, releases oxyHb, which promotes proinflammatory and thrombotic reactions. The presence of free oxyHb in plasma leads to a disruption of homeostasis. In response to free oxyHb, TF, the initiator of blood coagulation by macrophages, is expressed. However, the binding of oxyHb to TF leads to the weakening of oxyHb’s pro-oxidative properties [54]. Bacteria release many toxins, which can lead to vein thrombosis.

In addition to bacteria, also viruses are involved in thrombosis. For example, the influenza A virus (IAV) that infects the respiratory tract of humans leads to uncontrolled thrombosis, disseminated intravascular coagulation, and formation of pulmonary microembolisms with consequent haemorrhage and pulmonary oedema [55]. However, over-activation of the clotting system can cause lung damages and cardiovascular disease. For example, IAV infections lead to an increased risk of deep vein thrombosis and pulmonary embolism, acute coronary syndromes, and acute myocardial infarction [56]. In turn, influenza A virus subtype H1N1 increases the expression of genes promoting haemostasis, increasing platelet aggregation and platelet signature genes related to acute myocardial infarction (AMI) [57]. Dramatically increased PE associated with abnormal coagulation profile has been observed in patients with COVID-19. These patients had a significantly higher inflammatory syndrome, a more distal thrombosis process involving the deep vein system of the shin, and a higher disease severity score on computed tomography SARSCoV-2. The authors hypothesized that COVID-19 patients may have local pulmonary thrombosis rather than an embolism [58].

## 4. Regulation of Cells Activation in Development of VTE

### 4.1. Platelets Activation

A blood platelet is sensitive to factors from the surrounding environment via a contact with receptors present on its surface and takes part in the interactions of the platelets between each other and with other cells. Activation of platelets by inflammatory signals or infection results in P-selectin exposure and expression of cluster of differentiation-154 (CD-154) and CD-40 and leads to interactions with blood cells such as neutrophils, eosinophils, T-cells, B-cells, and ECs. Interactions with ECs lead to the expression of E-selectin and release of ICAM-1 and VCAM-1 adhesion molecules and to leukocyte recruitment [59]. Moreover, activation of platelet leads to exposure of glycoprotein (GP) IIb/IIIa, which is a receptor for fibrinogen. In turn, the GP Ib-IX-V complex is involved in the binding of platelet to the damaged epithelium via vWF. In contrast, GPs from the protease-activated receptor (PAR) family participate in platelet stimulation by thrombin. Other receptors associated with platelet activation include the thromboxane A2 (TXA2) receptor and platelet activating factor (PAF) receptor and the platelet C-type lectin-like receptor (CLEC-2). In homeostasis, GP Ia/IIa and GP VI also have a crucial role, being involved in the platelet response to collagen [60].

Activated platelets (PLa) release a number of active substances from the α and β granules, which additionally intensify their activation. The platelets also liberate serotonin, which causes narrowing of the blood vessels in the wound [13]. In addition, stimulated platelets release other adhesive GPs from granules such as fibrinogen; thrombospondin; vWF; coagulation factors: FV, FXI, FXIII, protein S; mitogenic factors: platelet-derived growth factor (PDGF), transforming growth factor beta (TGF-β), epidermal growth factor (EGF); angiogenic factors: vascular endothelial growth factor (VEGF), PF4 inhibitor; chemokines: chemokine CXC ligand (CXCL7), CXCL4, CHCL1, CXCL5, CCL5 (RANTES), CCL3 and cytokines: IL-1β, TGF-β, fibrinolysis inhibitors, plasminogen activator inhibitor-1 (PAI-I) [61].

Numerous experimental and clinical studies confirm the key role of ROS in the mechanism of platelet stimulation. There are many activation pathways, one of which is an increased isoprostane formation. The main source of superoxide and other ROS is NADPH oxidase 2 (NOX2) [62]. One source is the arachidonic acid (AA) metabolism and associated lipoxygenase (LOX) expression, the other is NADPH oxidase (NOX) [63]. ROS form within PLa and regulate their responses to collagen-mediated thrombus formation [64].

It was shown that a platelet can be strongly stimulated by advanced glycation end products (AGEs) that result from the non-enzymatic reaction of amino acids with sugars in pathological conditions such as diabetes and/or uraemia, but also during aging, which lead to expression of CD62 and CD63. In addition, AGEs induce the expression of blood TF. It was shown that high TF expression on monocytes and vascular endothelium regulates the inflammatory response. The adhesion of PLa to leukocytes has a key role in thrombus formation [65].

Additionally, inflammation initiated by pathogens also leads to the activation of blood platelets, if accompanied by damage to the endothelium, then fibrin builds up and a clot forms [66]. Bacteria can also release substances that activate platelets. One of them is gingipain, an enzyme released by *Porphyromonas gingivalis*, which activates the thrombin receptor on platelets [67]. In turn, STEC strains *Escherichia coli* releases and the Shiga toxin activates platelets through platelet glycosphingolipid [68].

In veins and larger arteries, the flow is fast enough that the blood behaves like a Newtonian fluid, and the shear stress generated is proportional to the shear rate [69]. In the formation of thrombosis, an important role is held by shear forces induced by blood flow, because they contribute to the excessive activation of platelets, their aggregation leading to the formation of a clot. Studies have shown that in clogged blood vessels, shear forces can be up to two orders of magnitude higher than in healthy vessels [70]. Additionally, shear stress leads to activation and alterations in vWF structure. High shear stress and associated platelet activation is responsible for venous and arterial thrombosis. At low shear stress, the main role is held by fibrinogen, while at high shear stress, the adhesion and aggregation of platelets is determined by vWF, but not fibrinogen [71]. Higher level of vWF is a risk factor in acute coronary syndromes, and patients with vWF deficiency show resistance to thrombosis [72,73]. In in vivo studies, a significant increase in vWF levels occurred below the site of coronary occlusion and endothelial damage, which led to increased platelet reactivity to high shear stress. In vitro tests have revealed markedly increased platelet activity by doubling the vWF concentration, which indicates a link between vWF concentration and platelet reactivity [74].

Platelet hyperactivity promotes the pathogenesis of cardiovascular disease and may be affected by elevated levels of circulating growth factors, which include insulin-like growth factor-1 (IGF-1). Although IGF-1 cannot initiate platelet activation, in combination with physiological stimuli, it can intensify this process through a phosphoinositide 3 dependent mechanism [75]. During sluggish blood flow, the levels of coagulation factors and thrombin are locally very high, which promotes platelet adhesion and aggregation and, consequently, leads to the formation of a blood clot [76,77].

Both infection and inflammation can directly or indirectly activate platelets through different receptors, triggering aggregation and thrombi formation within the vasculature. However, many reviews based on current literature summarize the impact of potential risk factors related to the involvement of platelets in the pathogenesis of VTE, as well as in predicting the risk of VTE events [78]. Several platelet protein polymorphisms have been found to be associated with VTE and are considered hereditary risk factors. These include polymorphisms of glycoproteins GPIa/IIa, GPIIb/IIIa and GPVI as well as the Janus kinase 2 (JAK-2) mutation. Additional risk factors may be expression markers such as tissue factor containing microparticles and P-selectin, which is a platelet activation marker, enhances microparticle production, tissue factor expression, and platelet aggregation. Additional factors of thrombosis and recurrent VTE in antiphospholipid syndrome (APS) are the increased mean platelets volume (MPV) and their increased number [79].

Various mechanisms of platelet activation, release of ROS by these cells as well as the involvement of platelets and vWF in clot formation have been described.

### 4.2. Endothelial Cells Activation

**Figure 3:**
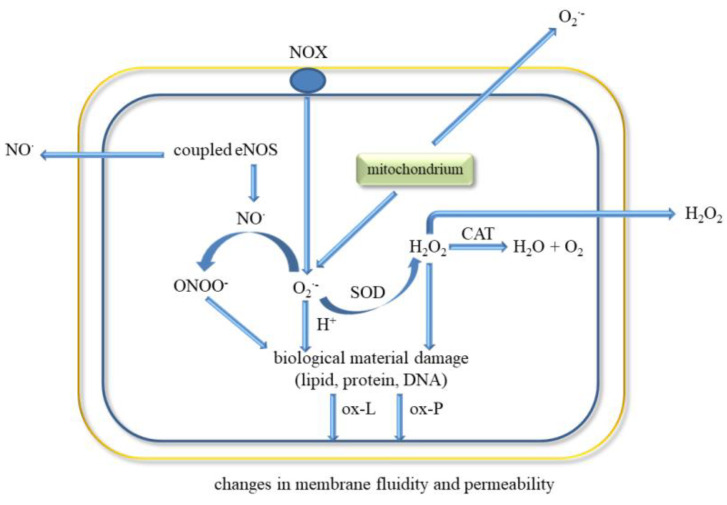
ROS release and oxidative biological material damage in endothelial cell. Abbreviations: catalase (CAT), water (H_2_O), hydrogen peroxide (H_2_O_2_), endothelial nitric oxide synthase (eNOS), nitric oxide (NO^•^), NADPH oxidase (NOX), oxygen (O_2_), superoxide anion radical (O_2_^•−^), peroxynitrite (ONOO^−^), oxidised lipids (ox-L), oxidised proteins (ox-P), superoxide dismutase (SOD).

Endothelial cells have a key role in homeostasis by having a direct contact between the blood and the vessel wall. They are involved in various functions such as regulation of blood flow, vascular permeability, recruitment of inflammatory cells, thrombosis, and angiogenesis. These cells secrete molecules that act on smooth muscle cells associated with vascular tone regulation and leukocytes that regulate inflammation state. ECs release prostacycline (PGI) and NO^•^, both substances that inhibit platelet adhesion and vasodilators (Figure 3) [8,80].

The classic factors leading to endothelium dysfunction include dyslipidaemia, smoking, hypertension diabetes mellitus, and aging. Other factors are infection inflammation, physical inactivity, high level of homocysteine, post prandial state, and obesity [80]. Excessive generation of ROS in the vascular wall is a common feature of many cardiovascular diseases (Figure 3). It was shown that EC-derived ROS have a key role in aortic wall susceptibility to dissection [81].

**Figure 4:**
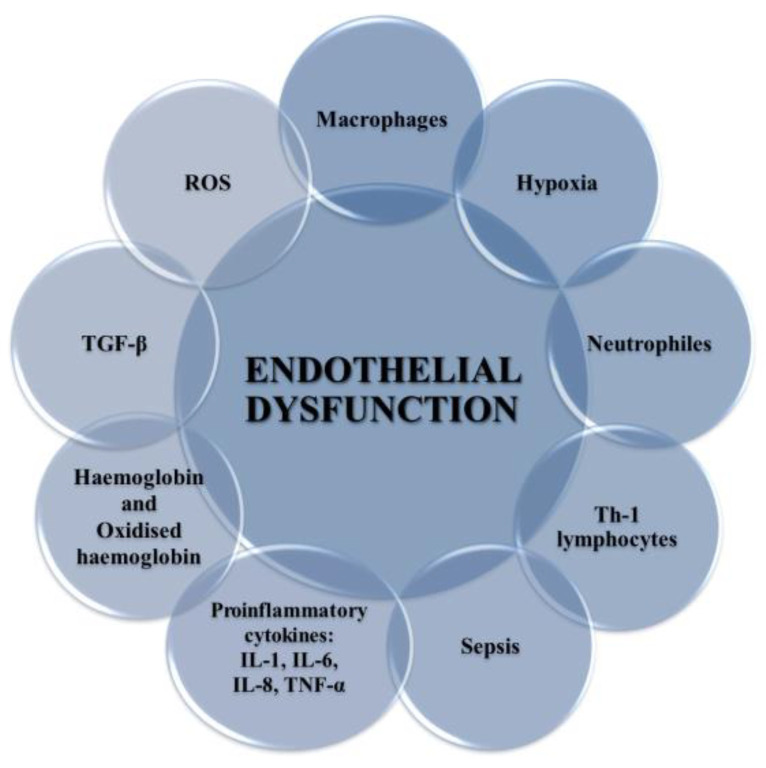
Factors leading to the development of endothelium dysfunction. These include ROS, proinflammatory cytokines (IL-1, IL-6, IF-8, TNF-α), TGF-β, Hb and oxyHb, Th-1 lymphocytes, sepsis, macrophages, and neutrophils.

Endothelial dysfunction occurs in both varicose veins and DVT [82]. It has been shown that ECs can be damaged by the endotoxin part of outer membrane of Gram-negative bacteria in bacterial pathogenesis and endotoxic shock (Figure 4) [83]. Activation of ECs is associated with increased expression of cellular adhesion molecules VCAM-1 and ICAM-1, which causes ED and promotes monocyte adhesion [84]. This process can be triggered by ischemia, catecholamines, angiotensin II, cytokines (IL-1, IL-6, TNF-α, TGF-β), and endotoxins (Figure 4). The presence of adhesive molecules leads to increased interaction between inflammatory cells and the endothelium and their migration into the endothelial space [37,85,86].

Stimulation of ECs leads to the release of microparticles of endothelial cells (EMP). Their presence in plasma has been found in many immuno-inflammatory diseases such as atherosclerosis, sepsis, and others [87]. Other agents released by endothelium include PAF, vWF, thrombomodulin, TF, t-PA, ectonucleotidases, and type I PAF [8]. 

**Figure 5:**
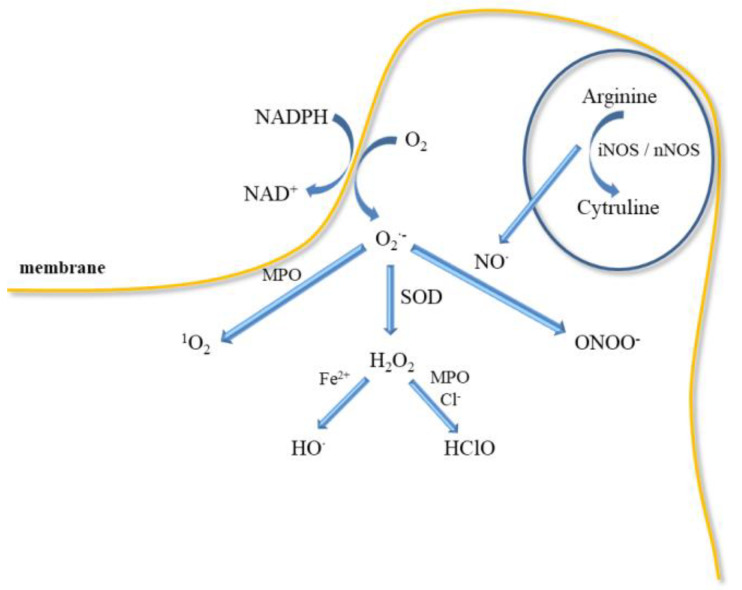
ROS can destroy phagocytized pathogens. Respiratory burst of neutrophil, which leads to the activation of NADPH-oxidase producing O_2_^•−^, precursor of other ROS, such as, H_2_O_2_, which can oxidize chloride to HClO in the presence of MPO, HO^•^ release in Fenton reaction.

Inflammatory mediators promote the adherence of neutrophils to the endothelium and their activation. Activated neutrophils, being inflammatory cells, as a result of respiratory burst release large amounts of ROS such as superoxide, H_2_O_2_, hydroxyl radical (HO^•^), nitric oxide, and hypochlorous acid (HClO) (Figure 5). ROS produced during inflammation lead to oxidative stress but are also used to remove pathogens [88]. In addition, ED leads to increased production of O_2_^•-^ and H_2_O_2_ catalysed by xanthine oxidase, which is accompanied by an increase in the synthesis of peroxynitrite, the factor responsible for damaging the biological material [43]. Activation of endothelial cells releases many active substances that may be involved in clot formation.

## 5. ROS and Oxidative Stress

The release of lower ROS levels by host cells is associated with their physiological function in regulating processes such as haemostasis, apoptosis, immune response, and microbial colonisation [46]. ROS are produced of numerous enzymatic reactions in various cell compartments, including the cytoplasm, cell membrane, endoplasmic reticulum, mitochondria, and reactions peroxisome as intra- and intercellular signalling molecules. The precursor of ROS is the superoxide anion radical (O_2_^•−^), which is formed as a result of a one-electron reduction of molecular oxygen. Superoxide anion radical is formed by the enzymatic process, autooxidation reaction, and by nonenzymatic electron transfer reactions in which an electron is transferred to molecular oxygen [89]. Superoxide is mostly produced within the mitochondria in electron transport chain during electron leakage. There are 11 sites in the respiratory chain in mammalian mitochondria where superoxide and/or hydrogen peroxide is formed [90]. Other enzymes that can produce superoxide are lipoxygenase, cyclooxygenase [91]. Superoxide can exist in two forms such as O_2_^•−^ or hydroperoxyl radical (HO_2_^•^) at low pH [92]. Its reactivity with the biomolecules is low while the reactivity of hydroperoxyl radical is much higher. Hydroperoxyl radical can penetrate the phospholipid bilayer easier than the charged form (O_2_^•−^). Polymorphonuclear leukocytes (PMNs), monocytes, and macrophages perform an important role in the innate immune response to pathogens. They can produce and release ROS in significant amounts [93]. One of the main sources of peroxide in phagocytic cells is membrane NAD(P)H oxidase [94]. Spontaneous or enzymatic dismutation of superoxide leads to hydrogen peroxide. Hydrogen peroxide can diffuse through the mitochondrial membranes into the cytoplasm [95]. The superoxide produced during phagocytosis is transformed into other ROS, which are directed to the absorbed material in the phagolysosome. Depending on the way used to activate the NAD(P)H oxidase, the production of superoxide can also target the extracellular environment [96]. ROS perform a physiological role as signalling molecules; however, their increased production and/or failure of antioxidative systems may lead to oxidative stress, which is associated with damage/modification of life-important molecules such as DNA, proteins, and lipids. It has recently been shown that certain molecules produced during the blood clotting process can stimulate PMNs to produce ROS. Released ROS by PMNs can damage the endothelium and affect the coagulation process [97].

The release of high ROS levels due to acute infection or inflammation may lead to oxidative damage to the host’s biological material. ROS have a crucial role in vascular function. Redox balance is important in cell signalling and haemostasis. However, perturbation of redox state leads to ROS interaction with important vital macromolecules such as proteins, enzymes, nucleic acids that can lead to vascular pathology [98,99]. ROS are associated with the pathogenesis of many diseases, including cardiovascular diseases such as hypertension, ischemic heart disease, ischemia-reperfusion injury, and other vascular diseases including thrombosis. ROS may also lead to other pathological conditions, such as pulmonary fibrosis and vascular retinopathy [37,100].

In pathological conditions, there is a continuous recruitment of inflammatory cells that release ROS [101]. This process is observed, for example, in atherosclerosis and a long-term venous disease. Excessive ROS formation and/or a decrease of antioxidant potential leads to changes in the vessel wall [102]. Eukaryotic cells have numerous organelles in which ROS can be produced. The largest amounts of ROS are released in the ETC in the mitochondria [103].

In the vascular system, ROS are generated mainly by NOX but also uncoupled NOS, xanthine oxidase, COX, and myeloperoxidase (MPO). NOX occurs in the isoforms NOX1, NOX2, NOX4, and NOX5. It was shown that the expression of isoforms 1, 2, 4 is the cause of cardiovascular diseases [100,104,105]. NOX4 participates in the production of oxygen active compounds through internal mitochondrial channels, including ATP-dependent potassium channels and mitochondrial permeability transition pore (mPTP) through which ROS can flow from the mitochondria to the cytosol [106,107]. NOX4 induced by TGF-β lead to ROS production, inhibition of mitogen-activated protein kinases (MAPK), MKP-1, and activation of RhoA [108]. NOX4 expression leads to DNA damage by H_2_O_2_, which was also shown diffused through nuclei membrane [109]. NOX can be enhanced by exogenous factors such as ultraviolet (UV) radiation, smoking, high calorie diets, and diabetes [100]. All these enzymes are complexes present in the membrane and are expressed in vascular tissue only to produce ROS. For example, NOX2 and NOX4 produce ROS in cardiomyocytes and fibroblasts, while NOX1, NOX4, and NOX5 do so in vascular smooth muscle cells (VSMCs) [108]. Other sources of ROS include peroxisomes, endoplasmic reticulum, and lysosomes, where they are produced by NOX, xanthine oxidases, cytochrome P450 (CYP), etc. [110].

However, large amounts of ROS are released by neutrophils during respiratory burst in response to infection because they have a crucial role in host defence and inflammation (Figure 5). Neutrophils participate in the immune responses associated with bacterial invasion, and they also produce nitric oxide, which is synthesised by neutrophilic inducible nitric oxide synthase. In addition to various physiological functions, their participation is associated with pathology [111]. Neutrophils may participate in numerous pathologies such as glomerulonephritis, diabetes, stroke, septic shock, whooping cough, sepsis, encephalitis, and ulcerative colitis. NO^•^ has been shown to mediate neutrophil extracellular traps (NETs) formation at the site of inflammation/infection by increasing ROS production. Activated neutrophils can form NETs, which include proteins, histones, chromatin, and DNA [90]. In turn, NETs increase coagulation by stimulating platelet aggregation, erythrocyte mobilization, and fibrin accumulation as well as endothelial activation and damage [112,113]. NO^•^ is also a mediator of neutrophil response in a neurodegenerative disease (Parkinson’s disease, schizophrenia, depression) and cardiovascular diseases, thrombosis, and hypertension [111,114]. Higher iNOS activity in neutrophils was observed in patients with congestive heart failure that positively correlated with elevated levels of IL-6 and noradrenaline in circulation [115].

The mechanism of bacteria removal by ROS is associated with an attack on membranes, but also with their penetration into the interior of bacteria, where they can damage nucleic acids, proteins, and enzymes [116]. However, NOX function is crucial for the formation of NETs [117]. NETs formation is also induced by proteases, which can be activated by ROS. Neutrophils can generate more ROS by responding to AGEs through the NOX complex [118,119]. Additionally, superoxide may be also released in mitochondria by NAD(P)H-oxidase, cytochrome c peroxidase or xanthine oxidase (Figure 5). Myeloperoxidase located in neutrophil granules can, with the participation of H_2_O_2_, oxidise chlorides to HClO, which is a strong oxidising agent and bacteria killing agent. MPO can also directly convert superoxide to singlet oxygen (^1^O_2_). Singlet oxygen can also be produced in a reaction of hypochlorite with hydrogen peroxide. However, the released H_2_O_2_ can be reduced in the presence of iron II to the HO^•^ (Fenton reaction). In addition, both ROS stimulate the production of proinflammatory cytokines as TNF-α and macrophage inflammatory protein 2 (MIP-2) [116,117,120].

Another important source of ROS in platelets are NOX occurring in various cell types, including ECs and other vascular cells. NOX expression can be triggered by hypoxia and endogenous factors, such as pro-inflammatory molecules, growth factors, PDGF, and hormones: angiotensin II and insulin [121,122]. ROS are also generated in the cells of VSMCs. This mechanism involves extracellular cyclophilin A (iCypA), which increases ROS production by angiotensin II [123].

Oxidative stress initiates the NF-κB transcription factor, which can cause the expression of AGEs and TNF-α [124]. Additionally, AGEs may promote oxidative stress which leads to the formation of oxidative protein products [125]. TNF-α is a pro-inflammatory cytokine that is released by active monocytes and macrophages and is associated with the inflammatory process in the host’s response to infection [126].

Proper functioning of the endothelium is also associated with the release of NO^•^ by endothelial nitric oxide synthase (eNOS) in which calcium ions participate [124]. Nitric oxide characterised by a low reactivity, free of electric charge, easily penetrates through the membranes of ECs and other cells. However, it can react with oxygen, yielding a strong oxidant and a nitrating agent, nitric dioxide [127]. In turn, the reaction of NO^•^ and the O_2_^•−^ leads to formation of ONOO^-^, which is characterised by similar properties as nitrogen dioxide, both inflammatory agents [128,129]. The constant rate of reaction of NO^•^ with O_2_^•−^ is about three times higher than dismutation of superoxide by superoxide dismutase (SOD) [130,131,132]. This supports the thesis that higher levels of superoxide and nitric oxide are harmful and in pathology lead to a damage to biological material [133]. In turn, oxyHb released in plasma can react with NO^•^ and produce ONOO^−^ and metHb [134,135]. It was shown that treatment of RBCs with nitric oxide led to metHb formation and oxidative damage of lipids and proteins in these cells [136]. It was reported that the lipid peroxidation product, 4-hydroxy-2-nonenal (HNE), causes changes in cryptic TF on the surface of blood macrophages and monocytic cell line, THP-1, converting it to TF procoagulant [137].

ROS affecting the processes of coagulation, fibrinolysis, proteolysis and complement, as well as the activation of platelets, endothelial cells, erythrocytes, neutrophils, monocytes may lead to the formation of a venous thrombus [138]. Furthermore, ROS can initiate coagulation inducing increased expression of tissue factor (TF) in endothelial cells, monocytes, and vascular smooth muscle cells, but NOX enzymes are necessary for ROS production [139,140,141]. It has recently been shown that certain molecules produced during the blood clotting process can stimulate PMNs to produce ROS. Released ROS by PMNs can damage the endothelium and affect the coagulation process [97].

This section describes the various sources of ROS and their effect on cell activation and thrombus formation as well as on damage to biological material.

## 6. Advances in Treatment of Thromboembolism

Conservative therapy is based on the administration of anticoagulants that reduce the risk of a pulmonary embolism. Anticoagulants have become the basis of DVT therapy and are used to prevent PE progression and recurrence of thrombosis. It was reported than the 30-day mortality risk for VTE patients was 3% for DVT not treated with anticoagulants. However, mortality risk increases to 31% in patients who have developed PE. In addition, anticoagulants inhibit the growth of existing thrombi and prevent the emergence of new ones. These medicines include low molecular weight heparin (LMWH), acenocoumarol, aspirin, and other antiplatelet inhibitors [142]. In addition, natural phlebotropic drugs such as rutin, aescin, pycnogenol, *Ruscus* or *Centella* extracts, *Ginkgo biloba,* and others strengthen and protect the walls of venous vessels [143]. 

The therapy uses oral anticoagulants for 3–6 months. Discontinuation of treatment in patients after the first episode of idiopathic VTE causes recurrent thrombosis with a frequency of approximately 5–10% in the first 2 years and in the following 1–3%. On the other hand, the long-term use of anticoagulants is associated with haemorrhagic complications. It is very important to clarify in which patients there is an increased risk of VTE recurrence. It is connected with the decision to extend the therapy and the isolation of the group of patients with low risk of VTE relapse, in whom anticoagulant therapy can be safely discontinued [144].

The variety of anticoagulants is associated with their use for the treatment and prevention different disease such as atrial fibrillation, deep vein thrombosis, pulmonary embolism, Ischemic stroke, myocardial infarction (heart attack), hip or knee replacement surgery, and others. Some drugs do not dissolve thrombi but prevent the growth of those already formed and the formation of new ones. In contrast, another group of drugs called thrombolytics (“clot busters”) can dissolve a clot during a period of a few days. 

### 6.1. Endovascular Treatment of DVT

Various methods are used to treat intravascular venous thrombosis, such as catheter-directed thrombolysis (CDT), catheter-directed pharmacomechanical thrombolysis (PCDT), percutaneous aspiration thrombectomy (PAT), vena cava filter protection, venous balloon dilatation, and venous stent implantation [145]. In addition, an intravascular PAT complementary technique is used, such as introducing a balloon to break up the thrombus, stent implantation and placement of a venous filter [145,146].

### 6.2. Anticoagulants

Drugs used to treat or prevent thrombosis can be divided into three groups: (1) anticoagulants that prevent the coagulation system and interfere with further plaque expansion, (2) antiplatelet agents that reduce platelet aggregation and inhibit blood clot formation, (3) fibrinolytic drugs that directly dissolve the resulting clot [147].

Under conditions of haemostasis, a balance is maintained through the interaction between platelets and vascular endothelium, which is also controlled by the cascade of roots and fibrinolysis. Continued cascade is associated with the transformation of prothrombin into thrombin, followed by the conversion of fibrinogen to fibrin, which forms a vascular clot with blood platelets (Figure 1). Additionally, thrombin activates other coagulation cascade factors like FV and FVIII, activates and releases FVIII from binding to vWF [148]. The standard pharmacological method of treating thrombosis is the use of anticoagulants, which reduce the formation of clots and prevent the further development of a thrombotic disease [149].

#### 6.2.1. Inhibitors of FX

In thromboembolic syndromes, coagulation FXa has a key role in the blood coagulation cascade. Factor Xa participates in both the external and internal coagulation pathways and is important in conversion of prothrombin to thrombin. The advantage of FXa inhibitors is the predictable anticoagulant effect and no need for routine monitoring. The introduction of selective inhibitors for FXa may reduce thrombin production causing an anticoagulant effect leading to smaller disturbances of primary haemostasis [150,151]. 

**Figure 6:**
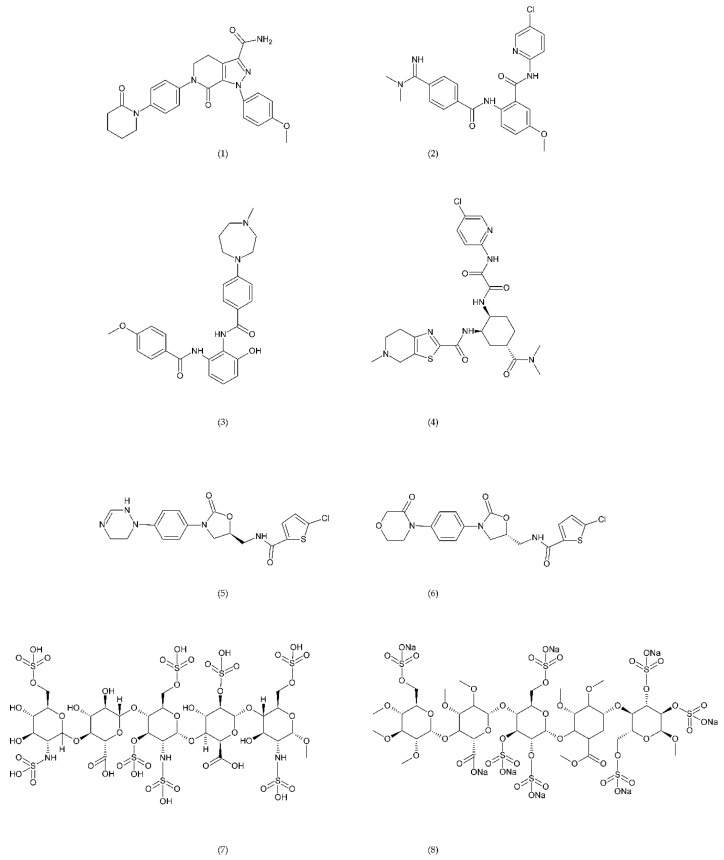
Direct factor Xa inhibitors: apixaban (1), betrixaban (2), darexaban (3), edoxaban (4), nokxaban (5), rivaroxaban (6), fondaparinux (7), and idraparinux (8).

A number of FX inhibitory drugs have been synthesised that have different effects. Their variety allows choosing the drug with the highest effectiveness and the smallest side effect depending on the type of disease and the patient’s condition [152]. Direct FXa inhibitors (Figure 6) may include: apixaban (1), betrixaban (2), darexaban (3), edoxaban (4), nokxaban (5), rivaroxaban (6) (BAY59-7939), and indirect FXa inhibitors: fondaparinux (7) and idraparinux (8) (antithrombin-binding pentasaccharide), dabigatran and LMWH. These drugs are used to reduce the risk of stroke and embolism in people with nonvalvular atrial fibrillation. They are also used to prevent deep vein thrombosis (DVT), which can lead to a pulmonary embolism (PE) in patients after a knee or hip surgery, as well as to treat DVT and PE, and to reduce the risk of both diseases coming back. Apixaban is metabolised by CYP mainly by CYP3A4, rivaroxaban by enzymes CYPA415, CYP2J2, in turn, nokxaban (GCC-4401C) by CYP2D6 and CYP2A4 [153,154,155]. 

Apixaban and other anticoagulants such as dabigatran, edoxaban, and rivaroxaban are effective, similar to warfarin in preventing haemorrhagic stroke in people with atrial fibrillation, and carry a lower risk of intracranial bleeding [156]. Apixaban is an anticoagulant medicine used to treat and prevent thrombosis, and it is also used to prevent stroke in patients with non-valvular atrial fibrillation. Apixaban is a drug that is more effective than aspirin or warfarin in reducing the risk of stroke and bleeding, reducing the mortality rate in this patient group. Apixaban, but also dabigatran or rivaroxaban, may be an effective alternative to warfarin in the prevention of stroke and thromboembolism, especially in women with paroxysmal or persistent atrial fibrillation [157]. However, patients should not have prosthetic heart valves, haemodynamic valvular disease, severe kidney failure, and advanced liver disease (impaired baseline clotting function). Factors such as individual risk of stroke and bleeding should be taken into consideration when choosing the appropriate anticoagulant, along with comorbidities, availability of agents that reverse the anticoagulant effect in the event of bleeding complications, international normalized ratio (INR) control possibility—a way of standardising prothrombin time (PT) measurement across labs [158]. Apixaban is also used in prevention of postoperative DVT in patients after total hip or knee arthroplasty [159].

Betrixaban, like apixaban, is used in the prevention of embolism following knee surgery and in the prevention of stroke following non-valvular atrial fibrillation and extended prophylaxis in high-VTE-risk [160].

Darexaban is an anticoagulant drug that is used in venous thromboembolism, after a major orthopaedic surgery, stroke in patients with atrial fibrillation, and in ischemic incidents in acute coronary syndrome [161,162]. Darexaban and its glucuronide or maleate specifically and competitively inhibit FXa. By inhibiting prothrombin at the sites where a blood clot (thrombus) forms, it leads to a reduction in the formation of blood clots in a dose-dependent manner [161]. Increasing the risk of myocardial infarction, unstable angina, venous thrombosis, and ischemic stroke [163].

Dabigatran (Dabigatran etexilate), an oral anticoagulant, is used in Europe and Canada for the prevention of stroke and secondary VTE following atrial fibrillation and joint replacement surgery [164]. 

Edoxaban is a medicine used to treat deep vein thrombosis and pulmonary embolism and to prevent the disease from coming back. Edoxaban is also used to prevent blood clots in patients with non-valvular atrial fibrillation who have at least one risk factor such as a history of stroke, high blood pressure, diabetes, heart failure, and age [165].

Rivaroxaban is a highly selective drug with a rapid onset of action that inhibits FXa by disrupting the internal and external pathways of the blood clotting cascade. In addition, it inhibits the formation of thrombin and the development of blood clots. This drug is used to prevent stroke and blood clots in patients with nonvalvular atrial fibrillation [156]. It is used together with aspirin to reduce the risk of heart attack and stroke in patients with coronary artery disease. This drug is also used to prevent VTE in some hospitalised patients who are at risk of blood clots due to reduced mobility and who do not have a high risk of bleeding. Rivaroxaban is associated with a lower frequency of severe and fatal bleeding than warfarin. However, rivaroxaban shows a higher rate of gastrointestinal bleeding [166].

Nokxaban is a new drug used to prevent VTE in patients who have undergone a hip or knee replacement surgery. An additional application of Nokxaban is the treatment of acute coronary syndromes for stroke prevention in patients with atrial fibrillation [167].

The risk of VTE with many anticoagulants has increased from low to high in the following order: rivaroxaban, apixaban, edoxaban, enoxaparin, darexaban, and betrixaban. In turn, the classification of clinically significant bleeding from low to high for the same drugs was as follows: betrixaban, enoxaparin, darexaban, edoxaban, apixaban, and rivaroxaban [168]. 

Another anticoagulant is c2 (NAPc2), an 85 amino acid peptide isolated from the nematode Ancylostoma caninum. NAPc2 binds to FX and FXa at a non-catalytic site and inhibits FVIIa. It has a half-life of almost 50 h, so it can be administered once every two days [169].

#### 6.2.2. Thrombin Inhibitors

Thrombin inhibitors are another group of drugs that are involved in thrombus formation. Thrombin activates FV, FVIII, and FXI, which participate in the release of more thrombin and activate FXIII, a protein involved in fibrin cross-linking and clot stabilisation. Thrombin also participates in the transformation of soluble fibrinogen into insoluble fibrin. Thrombin is a strong platelet agonist, and activated platelets allow the formation of clotting factor complexes that increase thrombin production by more than 1000-fold [170].

Inhibitors that inhibit free thrombin are commonly used anticoagulants, heparins such as unfractionated heparin (UFH) and LMWH (9). Molar mass of natural heparin chains ranges from 5000–40,000. In contrast, LMWH obtained in the 1980s contains chains with an average molecular weight of approx. 8000. UFH, or briefly heparin (10), is a naturally occurring glycosaminoglycan that binds to AT III, changes its conformation, and leads to its activation. 

The activated form of AT III causes strong inactivation of thrombin FIIa and FXa and to a lesser extent of IXa, XIa, and XIIa, preventing the formation of clots. In turn, LMWHs also show affinity for AT III and have a greater effect on FXa. Ultimately, both heparins inhibit thrombin activation. Heparin not only blocks the formation of fibrin but also inhibits the thrombin-induced activation of FV and FVIII [154,155].

**Figure 7:**
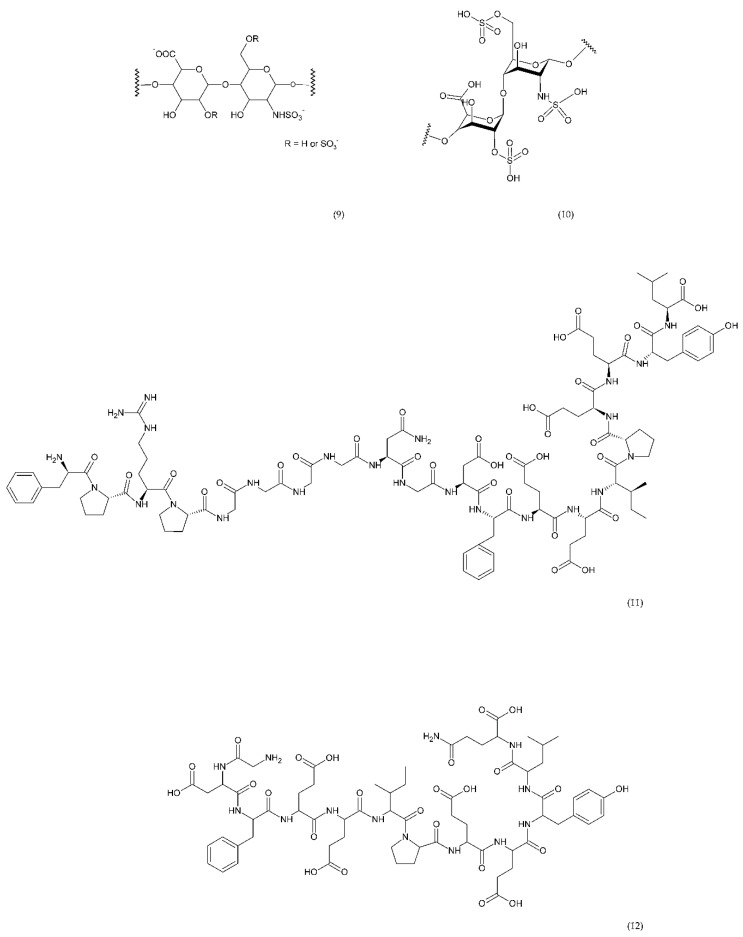
Direct bivalent thrombin inhibitors: low molecular weight heparin (LMWH) monomer structure (9), heparin (10), bivalirudin (11), and hirudin (12).

**Figure 8:**
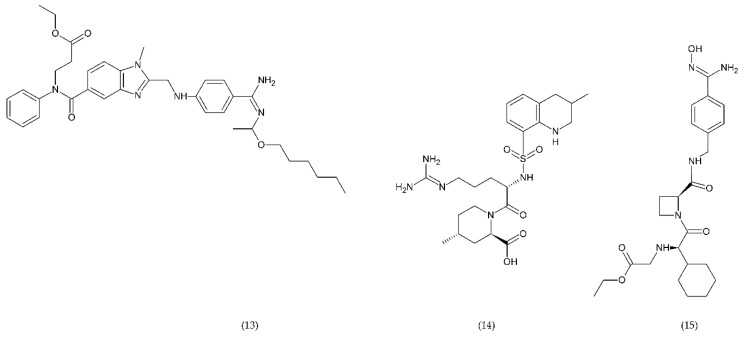
Direct monovalent thrombin inhibitors: dabigatran (13), argatroban (14), ximelagatran (15).

Direct thrombin inhibitors can be divided into bivalent, monovalent, and allosteric depending on their interaction with the thrombin molecule. Bivalent inhibitors such as bivalirudin (11) and hirudin (12) form a bond with the active centre and the external site of thrombin molecule (Figure 7). In contrast, monovalent inhibitors dabigatran (13), argatroban (14), and ximelagatran (15) bind only to the active site (Figure 8). In turn, allosteric inhibitors represent a third group that has been developed recently.

Hirudin is a natural protein isolated from leech’s saliva which has a significant anticoagulant effect and has been used in many countries as a natural anticoagulant [171,172]. The use of hirudin in combination with LMWH for perioperative treatment of senile intertrochanteric fractures for the prevention of DVT was more effective than LMWH-calcium alone. In addition, the drug combination did not affect the platelet count during treatment [173]. Direct thrombin inhibitors like bivalirudin are used instead of unfractionated heparin in in the treatment of thromboembolic events [174]. Bivalirudin is a synthetic congener of the naturally occurring drug hirudin. Bivalirudin is a dual inhibitor that inhibits collagen-dependent thrombin and platelet aggregation [175]. However, it has also been shown that bivalirudin blocks the endothelial bioavailability of NO^•^ and leads to vascular immobilization of MPO, a mediator of vascular function.

Dabigatran inhibits prothrombin but also inhibits the conversion of fibrinogen to fibrin. Table 1 presents the drugs used as anticoagulants in the treatment of thrombosis disease, the doses used and the mechanism of action. It has been shown that long-term use of Ximelagatran led to idiosyncratic liver toxicity and death, and it was withdrawn from the market [164]. 

#### 6.2.3. Other Compounds

**Figure 9:**
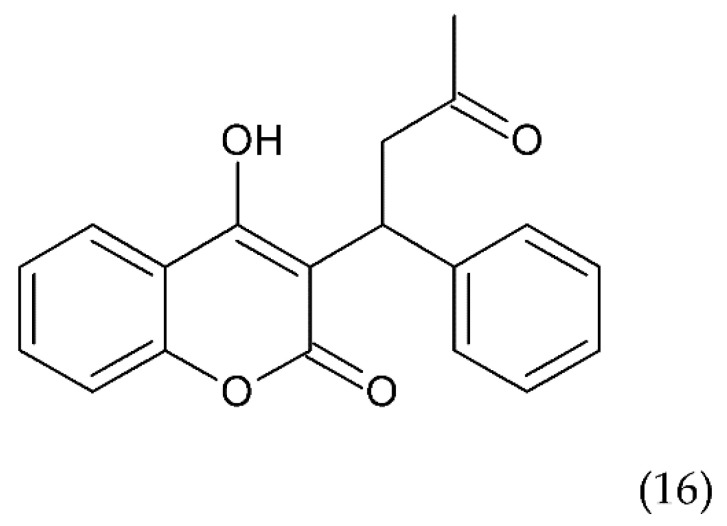
Chemical structure of warfarin (16).

Another drug commonly used to treat blood clots such as deep vein thrombosis and a pulmonary embolism is warfarin (16) (Figure 9), a coumarin derivative, which is an antagonist of vitamin K. Warfarin is an inhibitor of the vitamin K epoxide reductase complex (VKORC1), which performs a key role in activating vitamin K in the body. Warfarin lowers vitamin K reserves, leading to a reduction in the synthesis of the active clotting factors such as II, VII, IX, and X. Warfarin is used in medicine as an oral anticoagulant preventing stroke in people who have atrial fibrillation. It is also used in valvular heart disease or artificial heart valves and to prevent stroke in people who have atrial fibrillation, valvular heart disease or artificial heart valves. Warfarin is used for long-term anticoagulation or after a thrombotic event. It is also used to prevent thrombosis in high-risk patients, as well as in postoperative conditions. Warfarin combined with other anticoagulants is an effective medicine for the prevention of DVT in patients who have undergone DVT. It is also an effective drug in the prevention of DVT in cancer patients, especially those treated with chemotherapy [176].

Recently, a fully human antibody MAA868 was prepared. It binds to the catalytic domain of both forms of the enzyme FXI (zymogen) and the FXIa with high binding affinity. The safe and well-tolerated effects of single subcutaneous doses of MAA868 have been demonstrated in healthy subjects. MAA868 resulted in dose-dependent and time-dependent sustained prolongation of partial thromboplastin time after activation and FXI suppression to 4 weeks or more. Clinical trials have shown the possibility of a potential subcutaneous anticoagulant therapy once a month [177]. 

### 6.3. Antiplatelet Therapy

In thrombosis, pathological activation of platelets occurs, which in turn leads to uncontrolled clot growth, embolism, or blockage of the blood vessel. The corollary of this is organ ischemia. In the treatment of thrombosis, antiplatelet drugs (Table 2) are used, which include: COX-1 inhibitors, adenosine diphosphate (ADP) P2Y12 receptor antagonists, GP IIb/IIIa inhibitors, GP VI, GP IB/IX/V, prostaglandin E (PGE) synthase inhibitors, serotonin receptor 2A (5HT2A), PAR, and TXA2 inhibitors [191,192].

**Figure 10:**
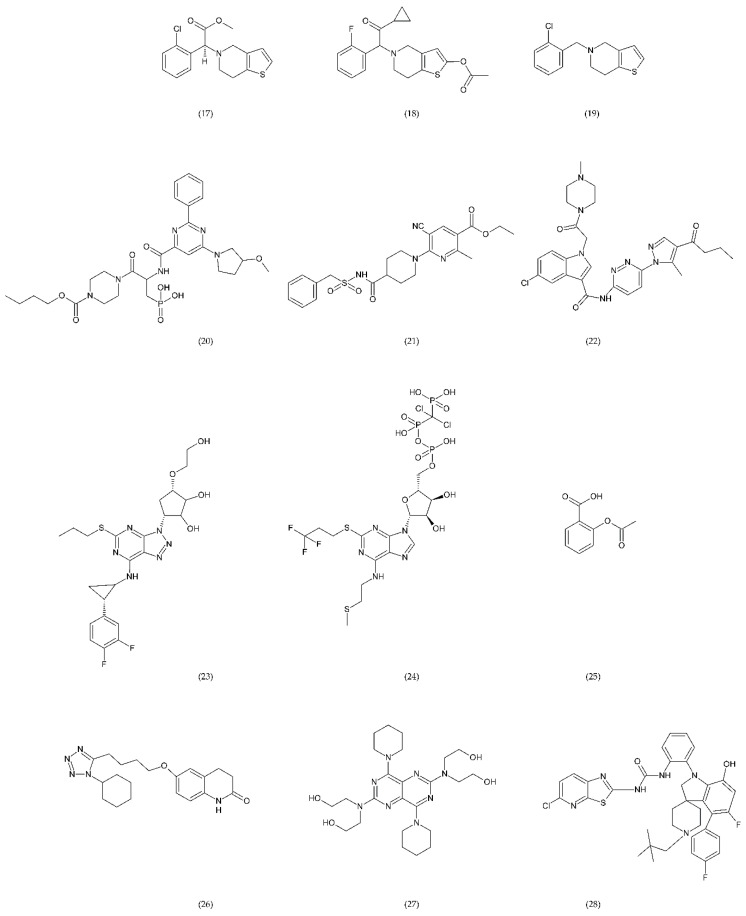
Antiplatelet drugs: clopidogrel (17), prasugrel (18), ticlopidine (19) as well as novel compounds ACT246475 (selatogrel) (20), AZD1283 (21), and SAR216471 (22) are active antiplatelet drugs irreversible, competitive, thienopyridine P2Y12 receptor antagonists, while ticagrelor (23) and cangrelor (24) are reversible P2Y12 receptor antagonists. ASA (25) inactivates cyclooxygenase (COX) by irreversible acetylation in the metabolism of arachidonic acid (AA). Cilostazol (26) inhibits the platelet aggregation induced by collagen, ADP, epinephrine, and AA. Dipyridamole (27) the potential of endogenous anticoagulants and the patency of small vessels and capillaries. BMS-884775 (28) and P2Y1 antagonist demonstrated similar anticoagulant properties such as prasugrel.

The most commonly used antiplatelet agents (Figure 10) include acetylsalicylic acid (ASA) and clopidogrel as well as dipyridamole and cilostazol as phosphodiesterase inhibitors [3,193,194]. Dipyridamole (27), antithrombotic drug, enhances the potential of endogenous anticoagulants and the patency of small vessels and capillaries, thereby preventing further tissue damage. In addition, it has a protective effect against oxidative stress [195]. Dipyridamole inhibits the release of proinflammatory cytokines and monocyte chemoattractant protein (MCP-1), matrix metalloproteinase (MMP-9), and leads to a decrease in high-sensitivity C-reactive protein (hsCRP) in patients [196].

Cilostazol (26) belongs to phosphodiesterase type 3 (PDE3) inhibitors, and its use causes an increase in cyclic adenosine monophosphate (cAMP). In turn, the increase in cAMP leads to an increase in protein kinase A (PKA), which directly affects the inhibition of platelet aggregation. In addition, PKA inhibits the activation of the myosin light chain kinase, which has an important role in the contraction of smooth muscle cells, which ultimately leads to vasodilatation [197]. It was shown that cilostazol was more effective in long-term use than aspirin and clopidogrel alone in patients with a previous ischemic stroke and had a significantly lower risk of bleeding. Cilostazol can be used in patients without heart failure to treat intermittent claudication [198].

ASA (25) inhibits platelet aggregation by inactivating COX through irreversible acetylation, which is involved in the metabolism of AA. Additionally, ASA inhibits the synthesis of MCP-1, inhibits the increased expression of matrix metalloproteinase-9, and attenuates the nuclear translocation of NF-κB [199].

Clopidogrel (17), prasugrel (18), and ticlopidine (19) are active antiplatelet drugs, irreversible, competitive, thienopyridine P2Y12 receptor antagonists. In contrast, ticagrelor (23), cangrelor (24), and ACT246475 (selatogrel) belong to the competitive reversible P2Y12 receptor blockade group. The P2Y12 receptor is G-protein coupled that binds ADP. ADP receptor binding results in inhibition of adenyl cyclase and cAMP secretion which leads to activation of the intracellular signal and alteration of glycoprotein conformation IIb/IIIa, enhancing fibrinogen affinity [200,201]. 

In addition to the P2Y12 inhibitors that initiate ADP-induced platelet aggregation in the study phase, there are other very potent inhibitors such as ACT246475 (20), AZD1283 (21), and SAR216471 (22) [202,203]. For example, ACT246475 undergoing phase II testing was characterised by less bleeding, higher selectivity, and ticagrelor-like anticoagulant activity. One of the methods used in anticoagulation therapy is blocking the P2Y12 receptor. In turn, P2Y1 antagonists show similar anticoagulant efficacy as P2Y12 inhibitors. BMS-884775 (28) is an antiplatelet drug, reversible and potent receptor antagonist of human P2Y1 receptors, it has good anticoagulant efficacy with less bleeding compared to prasugrel (P2Y12 antagonist) [202,204,205]. SAR216471 is a potent highly selective and reversible inhibitor, which has higher therapeutic index than clopidogrel, prasugrel, and ticagrelor [206]. AZD1283 inhibitor of P2Y12 has high potential almost 2-fold higher than ticagrelor and 4-fold higher than clopidogrel [206,207].

**Figure 11:**
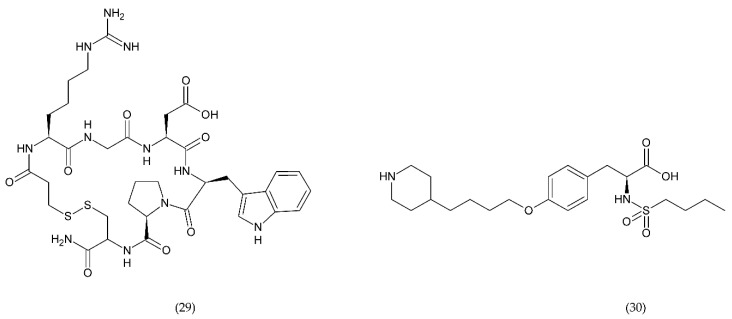
Antiplatelet drugs which selectively and reversibly bind to the platelet IIb/IIIa receptor glycoprotein: eptifibatide (29) and tirofiban (30).

Glycoprotein IIb/IIIa has an important role in the adhesion and aggregation of platelets and occurs quite abundantly on the surface of platelets being fibrinogen binding site. Eptifibatide (Figure 11) is a cyclic natural heptapeptide that inhibits platelet aggregation found in the venom of the rattlesnake, *Sistrurus miliarus barbouri*. Eptifibatide is characterised by its antithrombotic activity, which selectively and reversibly binds to the platelet GP IIb/IIIa receptors by blocking the binding of fibrinogen, vWF and other adhesive molecules [208].

Tirofiban (Figure 11) belongs to L-tyrosine derivatives in which the hydroxyl group in the ring is involved in the binding of piperidine residue, and in the amino group of the amino acid there is a butylsulfonyl residue. Tirofiban is an antiplatelet drug that inhibits aggregation of platelets and belongs to the reversible antagonists of GP IIb/IIIa receptors. The drug blocks the binding of fibrinogen molecules to GP IIb/IIIa receptors [192,208]. 

### 6.4. Emerging Drugs 

#### 6.4.1. RUC-1, RUC-2, and RUC-4

**Figure 12:**
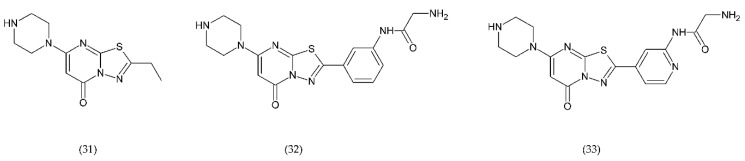
Chemical structures of RUC-1 (31), RUC-2 (32), RUC-4 (33).

In turn, the new drugs under study are RUC-1 (31), RUC-2 (32), and RUC-4 (33) (Figure 12), which are inhibitors of fibrinogen binding, specific for the α(IIb)β subunit of the GP IIb/IIIa receptor. These drugs, interacting with the receptor, prevent fibrinogen from binding. RUC-1 is an α(IIb)β inhibitor that binds selectively to the α(IIb) subunit. In contrast, RUC-2, which is a derivative of RUC-1, has approximately ~ 100-fold higher affinity for binding to the same subunit α(IIb) as RUC-1 [220]. RUC-2 additionally forms a bond with the β residue of metal ion-dependent adhesion site (MIDAS) of glutamic acid 220. In turn, RUC-4 showed approximately 20% greater inhibition of ADP-induced platelet aggregation than RUC-2. The reaction of RUC-4 with the receptor does not depend on whether it is in an active state or not, which is why it affects the entire platelet population. Both RUC-2 and RUC-4 have a unique mechanism of action consisting in blocking the receptor in its inactive conformation. In general, other drugs can lead to a conformational change and can contribute to thrombocytopenia in a small percentage of treated patients who have developed antibodies to the altered conformation [221,222].

#### 6.4.2. PAR-1 and PAR-4 Antagonist

**Figure 13:**
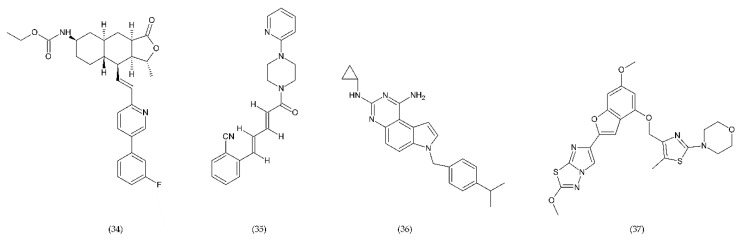
PAR-1 and PAR-4 antagonists: vorapaxar (34), F16618 (35), SCH79797 (36), BMS986120 (37).

Protease-activated receptors (PAR) have a special role in the development of chronic inflammatory diseases. The PAR family is conjugated to the G protein through the G domain. Activated receptors are involved in the transmission of signals by the recruitment of G proteins [223]. Thrombin, which is one of the key mediators of blood coagulation, activates a specific G-protein coupled receptor, called PAR-1. Vorapaxar (34) (himbacine analogue from *Galbulimima baccata*) is a drug that inhibits thrombin-mediated platelet activation (Figure 13). Its mechanism of action is related to the inhibition of protease-activated receptor-1. It is used in patients at high risk of ischemia, with an ischemic heart disease, and with a history of myocardial infarction. Moreover, it is characterized by a low risk of bleeding [224].

BMS986120 (37), which belongs to PAR4 antagonists, is a novel, selective and reversible drug that reduces the platelet aggregation during high shear stress [202,225].

A strong antithrombotic activity by F16618 (35), antagonist of PAR1, was demonstrated both in in vivo and ex vivo studies [226]. In addition, F16618 after oral or intravenous treatments reduced by 30 and 50%, respectively, the expression of the inflammatory cytokine TNF-α, 24 h after angioplasty [227].

In turn, SCH79797 (36), the PAR1 antagonist, was effective in reducing thrombi of fibrin-rich platelets at high shear rates (Figure 11). SCH79797 studies on experimental animals have shown a high potential for PAR1 antagonism in the treatment of diseases such as vascular thrombosis [228]. The antithrombotic effect of SCH79797 was significantly increased in combination with aspirin [229].

#### 6.4.3. Inhibitors of PDI and PI3Kβ

In the process of haemostasis and thrombosis, the PI3Kβ/protein kinase B (Akt) signalling pathway associated with platelet activation and aggregation is important. The family of phosphoinositide 3-kinases (PI3Ks), which catalyse the phosphorylation of the inositol ring of phosphatidylinositol, acts as regulator of cellular function supporting platelet activation and thrombus formation. PI3K enzymes are also involved in cardiovascular diseases including angiogenesis, hypertension, and heart failure, and are the target of promising treatment for the prevention of thrombosis [219].

**Figure 14:**
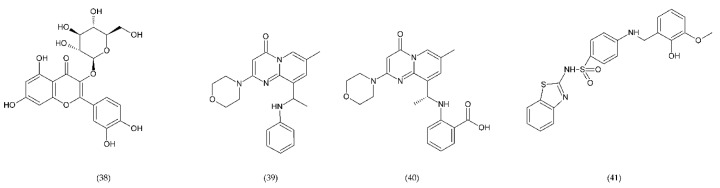
Inhibitors of PDI and PI3Kβ: Isoquercetin (38), TGX221 (39), and AZD6482 (40); inhibitor of 12-lipoxygenase ML355 (41).

The use of drugs that inhibit this pathway can be a method of treating thrombosis in patients with implanted blood contacting medical devices [230]. A promising anticoagulant is isoquercetin (38), inhibitor of PDI found on the surface of PLa. Preliminary studies using this flavonoid have shown a reduction in platelet aggregation and limitation of thrombus formation. Isoquercetin reduces platelet-dependent thrombin production by blocking the production of platelet factor (PF) Va (Figure 14) [202].

TGX221 (39) related pyrido[1, 2-a]pyrimidin-4-one belongs to a new target for antithrombotic therapy, and it is a PI3Kβ inhibitor. In platelets, PI3Kβ is an effector of various cell surface receptors including GP Ib, GP VI, and P2Y12. TGX221 is a potent and selective inhibitor [231]. The TGX-221 p110β inhibitor blocked IGF-1 initiated phosphorylation of Akt in platelets deficient in p110α, indicating that both p110α and p110β were involved in IGF-1 mediated phosphorylation of Akt [75]. Additionally, TGX221 has a particular role in shear stress conditions, becoming a new anticoagulant drug aimed at PI3Kβ [232]. In vivo studies have shown that inhibition of PI3Kβ provides protection against arterial thrombosis, with a limited effect on normal haemostasis [233].

AZD6482 (40), is an improved structural analogue of TGX-221 and is also PI3Kβ inhibitor that underwent preclinical and early clinical examination (phase I). AZD6482 exhibited antithrombotic effects without any increase in bleeding time [234,235]. AZD6482 inhibited agonist-induced platelet aggregation and shearing, showing concentration-dependent anticoagulation in vivo in dogs, with no detectable increase in bleeding time or blood loss [219]. Research conducted on healthy volunteers showed that the properties of the drug in inhibiting platelet activation placed it at levels between aspirin and clopidogrel. However, the maximum antiplatelet effect was evaluated clinically in combination of AZD6482 with ASA and clopidogrel [202].

ML355 (41) is a drug that inhibits platelet aggregation in humans. ML355 is a selective inhibitor of platelet 12-LOX, an enzyme in the AA pathway that leads to the formation of 12-HPET (Figure 14). It does not affect the activity of COX-1 and COX-2. ML355 inhibited dose-dependent aggregation of human platelets. ML355 treatment impaired thrombus growth in an arteriole in the mouse model [236,237].

#### 6.4.4. Other Compounds

During trauma and/or high shear stress, the GP Ib/IX/V receptor, which binds to vWF, has a key role, resulting in platelet adhesion to the endothelium. The drug that binds the A1 domain of vWF is aptamer ARC1779, 40-mer modified DNA/RNA oligonucleotide. Additionally, ARC1779 is the ligand for GP Ib receptor on platelets [238,239]. In people with acute myocardial infarction, the level of vWF was 2 times higher than in healthy people. ARC1779 blocks binding of the vWF A1 domain to GP Ib receptors. Aptamer inhibited vWF activity and shear-dependent platelet function. Unlike GP IIb/IIIa antagonists, ARC1779 did not inhibit platelet aggregation induced by ADP, collagen, or AA [240]. Compared with abciximab, ARC1779 showed comparable anticoagulant efficacy, however, with less prolonged bleeding time.

In a partial phase I/II clinical trial, ARC1779 used in patients with congenital thrombocytopenic purpura (TTP) inhibited vWF-induced platelet aggregation and stabilized platelet counts without any associated bleeding [241].

The next anticoagulant was the AJvW-2n monoclonal antibody directed against human vWF A1 domain, which inhibited platelet aggregation induced by high shear stress in vitro. Antibody also counteracted clot formation in vivo in photochemically induced thrombosis in the carotid artery of guinea pigs without affecting bleeding time [242].

Another aptamer of the second generation inhibiting vWF activity, as a result of shearing forces, and as a consequence suppressing plate aggregation, was ARC15015. This compound inhibited platelet adhesion stimulated in whole blood by ristocetin at 90% in the aortic segment under strong shear conditions [243].

A promising drug seems to be the humanised, bivalent Caplacizumab nanobody (ALX-0081) that binds to GP Iba on vWF. ALX-0081 has been approved by FDA in the treatment of adult patients with TTP [244,245]. The bivalency of ALX-0081 results in higher affinity for vWF. In vitro studies in the perfusion chamber using blood of healthy individuals and patients after percutaneous coronary intervention (PCI) receiving a standard therapy (aspirin, clopidogrel, and unfractionated heparin) showed inhibition of platelet adhesion to type III collagen by Caplacizumab under arterial shear conditions (high shear stress) without side effects under low shear conditions. Caplacizumab has recently been approved and is a new drug for the treatment of TTP. This drug specifically binds to the A1 domain of vWF, which blocks vWF from binding to platelets. Caplacizumab is the first drug in its class to show significant efficacy in the treatment of TTP and its complications in clinical trials [244].

Another vWF-binding drug, which is also a GP Iba antagonist, is the snake venom anfibatide of *Agkistrodon acutus*, which inhibits the interaction between vWF and PLa and consequently blocks platelet adhesion and aggregation [246]. In turn, the tripeptide of the isolated venom *Agkistrodon acutus* successfully inhibited the binding of fibrinogen to GP IIb/IIIa [247]. In addition, anfibatide had also a protective effect on cerebral ischaemia and reperfusion injury and its use could be beneficial for the treatment of ischaemic stroke [248,249]. Anfibatide is currently undergoing a clinical trial in acquired TTP which ends in 2021.

Recently, a new therapeutic antibody against platelet GP VI, ACT017, has been shown to have a strong and selective antiplatelet activity. GP VI present on the surface of the platelets is a physiological collagen receptor. The interaction of collagen with the GP VI receptor leads to the release of ADP and TXA2, which in turn causes the activation and adhesion of platelets and the activation of the GP IIb/IIIa receptor. Studies in healthy volunteers have shown that antibody ACT017 is well tolerated and safe to use [250]. Despite the wide variety of drugs used in thrombosis, they are characterised by bleeding risk, cardiovascular complications, and mortality. The introduction of new stronger antiplatelet agents is associated with an increased risk of bleeding. In vivo research related to haemostasis and thrombosis has revealed significant differences between these phenomena that have been helpful in developing new antiplatelet drugs that would not lead to increased bleeding. Preclinical therapies have been introduced in preclinical studies that inhibit thrombosis but do not affect the haemostasis process. This group of drugs includes PI3Kβ inhibitors, protein disulphide isomerase, activated GP IIb/IIIa, receptors activated by protease, and platelet-mediated adhesion pathways [192].

An interesting method to reduce the possibility of vascular thrombus formation is the use of CX3C chemokine receptor 1 (CX3CR1) and fractalkine (CX3CL1) receptor inhibitors. Monocyte-endothelial cells interactions are partly mediated by expression of monocyte CX3CR1 protein and endothelial cell CX3CL1. CX3CL1 is a membrane protein synthesized in ECs. ECs produce CX3CL1 after stimulation with TNF-α, IL-1, IFN-γ, and LPS. It was shown than the weakening of interaction between the pair CX3CL1-CX3CR1 receptor can reduce monocyte binding to the endothelium [251,252,253].

A new strategy in the treatment of thrombosis is to use FXI and FXII inhibitors. Both factors are important in thrombosis but not in haemostasis. Factor FXI has a much more important role in thrombosis than FXII. In addition, the anti-FXI antibody inhibits the activation of platelets and fibrin deposition more than in the case of FXII [254]. However, FXII may be a possible, therapeutic, and safe target to reduce platelet adhesion and fibrin formation during medical interventions [255]. One of the methods of action is to inhibit FXI by antisense oligonucleotides (ASO), which inhibit FXI synthesis in liver [256]. In turn, monoclonal antibodies may block FXI activation or FXIa activity [177,257], whereas aptamers binding to FXI or FXIa and small molecules block the active site of FXIa or/and induce allosteric alterations [258,259].

This section summarises the various anticoagulants such as FX inhibitors, thrombin inhibitors, antiplatelet drugs, emerging drugs, PAR antagonists, and PDI and PI3K inhibitors as well as other active compounds.

## 7. Natural Compounds

There is an abundance of drugs used in thrombosis; however, they lead to non-desirable side effects, mainly bleeding. The increase in cardiovascular incidents requires the introduction of new drugs that would be effective in the treatment and prevention of thrombosis and at the same time be free of side effects. Many plant compounds such as saponins, flavonoids, and others have an important role in preventing various diseases. Oxidative stress promotes thrombotic complications. Therefore, various substances of natural origin reduce the level of oxidative stress and may be important in the treatment of thrombotic complications [260]. Inactivation of ROS and alleviation of oxidative stress can reduce the risk of platelet hyperactivation, which leads to cardiovascular diseases including thrombosis [261]. Numerous works have shown that compounds of natural origin can reduce the risk of cardiovascular diseases including thrombosis. In Far Eastern medicine, medicines of vegetable origin have been used that have good healing properties and no side effects. These substances affect the mechanisms of thrombosis in various ways, affecting the coagulation system and platelet activation and aggregation and changing blood rheological conditions.

Drugs used in anticoagulation therapy with anticoagulation, inhibiting platelet aggregation and associated with fibrinolysis, have been presented.

### 7.1. Flavonoids

Natural products such as dietary supplements are important in ensuring health but have an important role in the prevention of cardiovascular and other diseases. Flavonoids as antioxidants reduce ROS levels and thus inhibit platelet aggregation/function or prothrombotic effects [262]. Flavonoids can alleviate ED, which is one of the factors leading to thrombosis, by inhibiting the excessive availability of TF in the endothelium [261]. Flavonoids can inhibit platelet activity by inhibiting the AA pathway [263] or may bind to the TXA2 platelet agonist and this way inhibit their aggregation.

**Figure 15:**
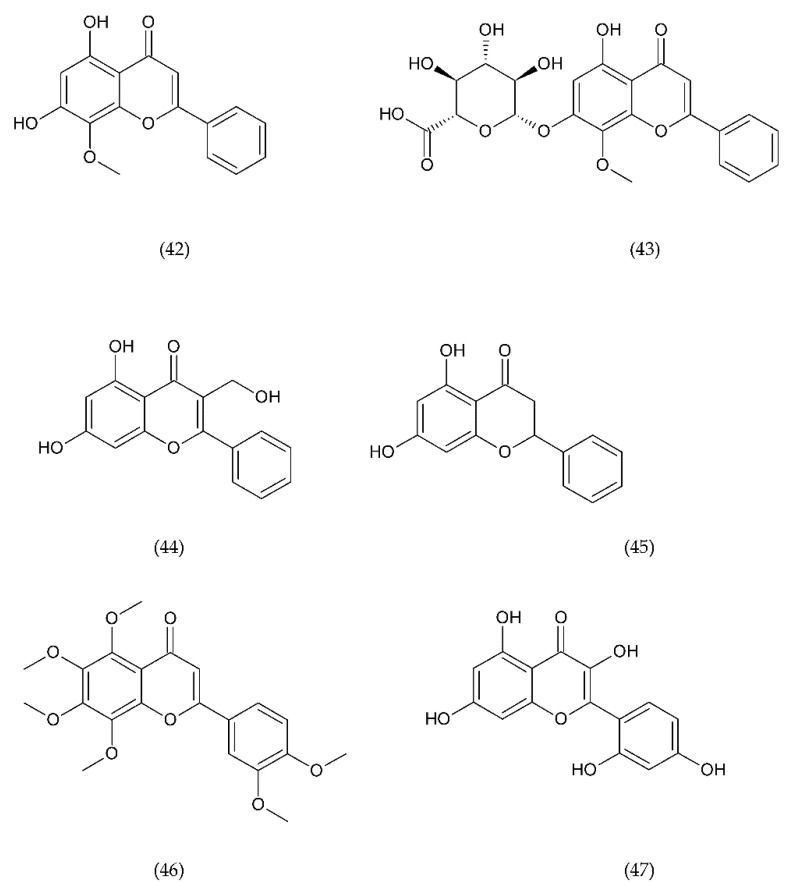
Chemical structures of flavonoids: wogonine (42) wogonoside (43), galangin (44), pinocembrin (45), nobiletin (46), and morin (47).

Natural dietary supplements including flavonoids are widely distributed in the plant world, especially in a variety of fruits, coffee, tea, cocoa, legumes, and other plant foods. The most popular flavonoids, including quercetin and its derivatives, and tea flavonoids, such as catechin, epictechin, epigallocatechin, and epigallocatechin gallate, have antioxidant, anti-inflammatory, anti-cancer, and anticoagulant properties, and are found in green tea [264]. But other less popular flavonoids have such properties as well like wogonine (42) from *Scutellaria baicalensis* Georgi and flavonoid wogonoside (43), which is a metabolite of wogonine isolated from *Scutellaria baicalensis* (Figure 15) [265].

#### 7.1.1. Antithrombotic Properties of Flavonoids

Anticoagulant activity associated with inhibition of platelet aggregation initiated by AA, ADP, and collagen as well as elongation of activated partial thromboplastin time (aPTT), PT, was shown using the extract of persimmon (*Diospyros kaki*) leaves, whose main ingredients are flavonoids such as catechins, epicatechin, and epicatechin gallate [266,267]. Similar results, associated with inhibition of platelet aggregation, were obtained using propolis, whose main ingredients are caffeic acid and its phenethyl ester, ferrulic acid; galangin (44); apigenin; quercetin; kaempferol; rutin; chrysin; pinostrobin; and pinocembrin (45) (Figure 15). The process of inhibiting platelet aggregation was induced by ADP, thrombin receptor activator peptide (TRAP), and collagen and was dose-dependent, which led to inhibition of thrombus formation [268].

Another flavonoid (Figure 15) with anti-inflammatory, antioxidant, anticancer, and anti-platelet inhibiting properties was nobiletin (46), a polymethoxylated flavone isolated from citrus fruit. Its mechanism of action is associated with inhibition of phosphorylation of phospholipase Cγ2 (PLCγ2), protein kinase C (PKC), Akt in mitogen stimulated MAPK protein kinase in collagen activated human platelets. In addition, nobiletin significantly reduced intracellular calcium mobilisation and hydroxyl radical generation, ultimately leading to inhibiting platelet activation [269].

The use of wogonins and wogonoside led to inhibition of thrombin and FXa activity and inhibited thrombin production in human umbilical vein endothelial cells (HUVEC), as well as resulted in prolonged aPTT and PT. Furthermore, both flavonoids inhibited thrombin-catalysed fibrin polymerisation and platelet aggregation and showed anticoagulant activity in vivo in mice. Wogonin and wogonoside reduced the ratio of PAI-1 to t-PA. These properties testify to the good anticoagulant effect of both flavonoids [270].

Flavonoids and their derivatives inhibited thrombus formation in vivo. For example, pentamethyl quercetin (PMQ), a methylated quercetin derivative, inhibited thrombus formation in mice with acute pulmonary thrombosis, leading to 90% of the mice surviving. It has also been shown that PMQ significantly improved blood flow in mice with damaged carotid artery showing an inhibitory effect on thrombus formation [271].

Diosmin (diosmetin 7-O-rutinoside), a disaccharide derivative that consists of diosmetin is a natural flavonoid found in the *Rutaceae* family or the *Citrus* family. Diosmin has the properties of an antioxidant, antihyperglycemic, anti-inflammatory, antimutagenic, and antiulcer agent and is commonly used to treat varicose vein [143]. Diosmin isolated from *Galium verum* also showed anticoagulant properties, and its mechanism of action was associated with regulation of centrosome-associated protein 350 (CEP 350), which is related to spindle assembly. Its use led to the improvement of venous thrombosis [272].

Another bioactive flavonoid with anticoagulant properties is morin. Morin (47) belongs to the flavonol group and is found in old fustic (*Chlorophora tinctoria*), osage orange (*Maclura pomifera*), almond, mill (*Prunus dulcis*), fig (*Chlorophora tinctoria*), onion, and apple. This flavonoid is a compound that prevents inflammation and apoptosis of cells and also has antioxidant activity (inhibits xanthine oxidase activity). Morin significantly inhibited human platelet aggregation stimulated by collagen, but not stimulated by other agonists. In collagen PLa, morin was shown to inhibit ATP release, intracellular Ca^2+^ ions mobilisation, P-selectin expression, PLCγ2 phosphorylation, PKC, and Akt [273].

**Figure 16:**
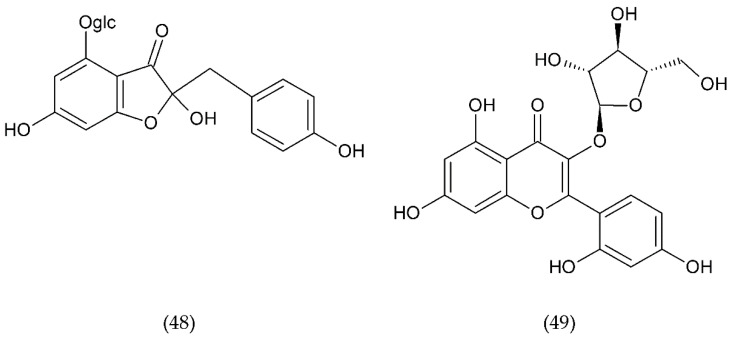
Chemical structure of hovertrichozide (48) and avicularin (49).

The anticoagulant properties of the extract from *Chaenomeles sinensis* have been shown. Thirteen flavonoids were isolated from fruit of *Chaenomeles sinensis*, and five of them, including hovertrichozide (48), luteolin-7-O-β-D-glucuronide, hyperin (quercetin-3-O-galactoside), avicularin (49) (quercetin 3-alpha-L-arabinofuranoside) (Figure 16), and quercitrin, were active in inhibiting TF expression induced by calcium ions in rat plasma in vitro. The activity of the hovertrichozide benzofuran derivative was higher than that of classic flavonoid derivatives of chroman, such as luteolin-7-O-β-D-glucuronide, hyperin, avicularin, and quercitrin [274].

#### 7.1.2. Isoflavonoids

**Figure 17:**
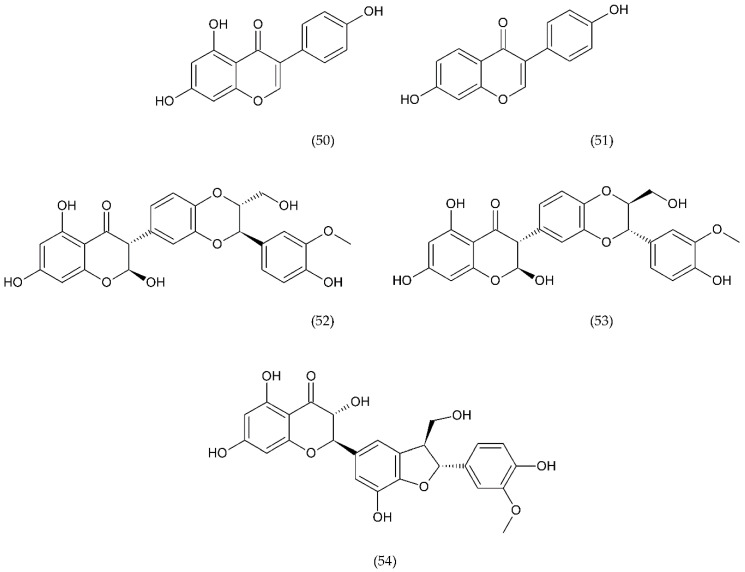
Chemical structure of isoflavonoids: genistein (50), daidzein (51), silybin A (52), silybin B (53), and silychristine (54) flavonoid.

In addition to flavonoids, isoflavonoids (Figure 17) also show antiplatelet activity. Isoflavonoids include genistein (50) and daidzein (51), which are COX-1 inhibitors and functional TXA2 receptor antagonists, affecting the platelet aggregation cascade. COX-1 inhibition by these isoflavonoids is similar to that of acetylsalicylic acid. The use of both isoflavonoids may have a positive inhibitory effect on the aggregation of human platelets in cardiovascular diseases in which they are associated with increased platelet activity [275].

Silymarin is the mixture of substances derived from *Silybum marianum* seed that exhibits antioxidant, anticancer, anti-inflammatory, and antiviral properties [276]. Silymarin inhibits cell proliferation and induces apoptosis, while also having anti-angiogenic properties. Silymarin consists mainly of silybin A (52) and silybin B (53), and other compounds. Both isomers of flavonoids belong to the group of flavonolignans. Silybin has been shown to be a signal transducer and activator of transcription 3 (STAT3) protein inhibitor whose function is to control the expression of genes that are involved in cell survival, proliferation, chemo-resistance, and angiogenesis. Silybin is also an inhibitor of expression of the programmed death-ligand 1 (PD-L1) checkpoint immune regulator as well as epithelial-mesenchymal transition (EMT) regulators, and it can be a promising adjuvant in non-small-cell lung carcinoma (NSCLC) [277].

Another flavonolignan, silychristine (54), is a 1-benzofuran derivative isolated from *Silybum marianum* or from silymarin. Silychristine also has antioxidant properties and is an inhibitor of lipid peroxidation in vivo and in vitro, LOX, and prostaglandin synthetase [276]. Silybin and silychristine inhibited collagen-initiated platelet activation. Significant decreases in platelet aggregation and microparticle aggregation were observed, as well as reduced expression of P-selectin and activation of αIIbβ3 integrin but also reduced adhesion and secretion of PF-4. Both can be used to prevent primary and secondary thrombotic events [278]. Strong inhibitors effects of silybin and silychristine on platelet activation induced by ADP have been shown [279].

This chapter discusses the anticoagulant mechanisms of action of popular flavonoids and isoflavonoids.

#### 7.1.3. Traditional Korean and Chinese Medicine

**Figure 18:**
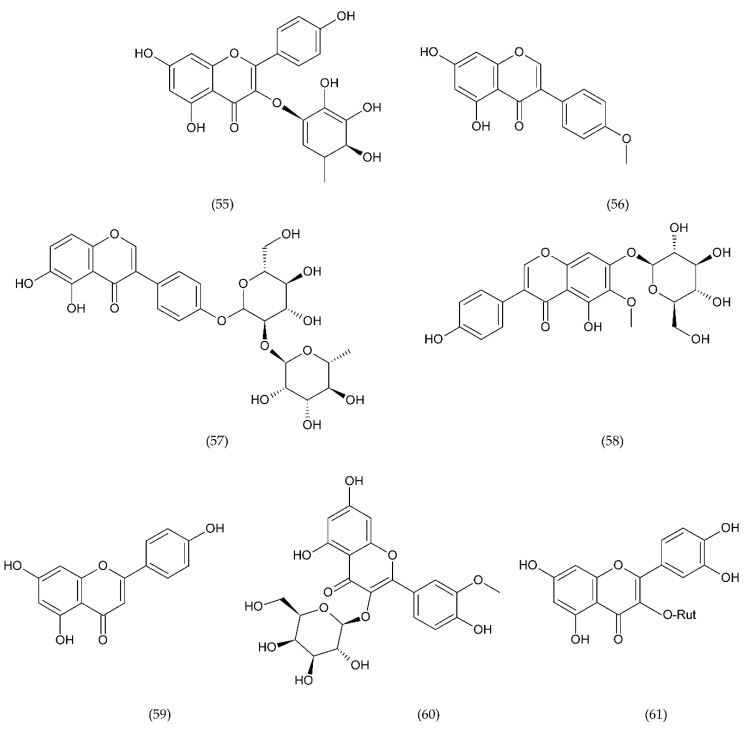
Chemical structure of afzelin (55), biochanine A (56), sophorabioside (57), tatectoridin (58), apigenin (59), isorhamnetin glucosides (60), and rutin (61).

*Lindera obtusiloba* (LLE) is used in traditional Korean and Chinese medicine for the treatment of blood stasis and inflammation. Additionally, the leaf extract showed various physiological effects. The main flavonoids in the extract are afzelin (55) (Figure 18) and quercetrin. Leaf extract was shown to significantly inhibit agonist-induced platelet aggregation in vitro and ex vivo and to inhibit collagen-induced TXA2 production in blood platelets. In addition, in vivo, the extract showed a protective effect on clot formation induced by intravenous injection of a collagen and adrenaline mixture. Further studies showed that the extract did not significantly affect PT and aPTT [280].

Buds and fruits of *Sophora japonica* (Leguminosae) plants widely distributed in Korea, China, and Japan have been used as haemostatic agents in traditional Korean medicine. *Sophora japonica* extract has antiplatelet activity. Four flavonoids and six flavonoid-glycosides were isolated from the extract, such as: biochanine A (56), eriodictyol, genistein, taxifoline, sophorabioside (57), genistin, tatectoridin (58), apigenin (59), quercetin, kaempferol, and its 3-O-rutinoside and 8-metoxykaempferol, isorhamnetin glucosides (60), rutin (61), and six derivatives of quercetin (Figure 18) [281].

### 7.2. Saponins

New natural substances with anticoagulant properties are currently being sought that would be free of side effects such as bleeding that may occur when using classic anticoagulants. Natural anticoagulants occur in the world of plants, which are traditionally used in Chinese medicine, but they also may be substances of animal origin [282]. The group of natural compounds with anticoagulant properties includes saponins, which occur in the world of plants and are produced by some marine organisms, including star fish and sea cucumbers. Saponins belong to amphipathic glycosides, which have one or more hydrophilic glycosides linked to a lipophilic triterpene or a steroid derivative [283]. Molecular weight depending on the basic backbone of the saponins and the number of sugar residues. We have corrected the molecular weight of saponins. Saponins have a high molecular weight ranging from 741 to 1808 g/mol [284].

#### 7.2.1. Plant to the Genus *Panax*

Ginseng is a plant belonging to the genus *Panax*, which has long been known for its medicinal properties that help alleviate disease symptoms and prevent potential diseases. Ginseng contains many bioactive components, mainly saponins, most of which are glycosides of triterpenoid aglycons, also known as ginsenosides [285].

**Figure 19:**
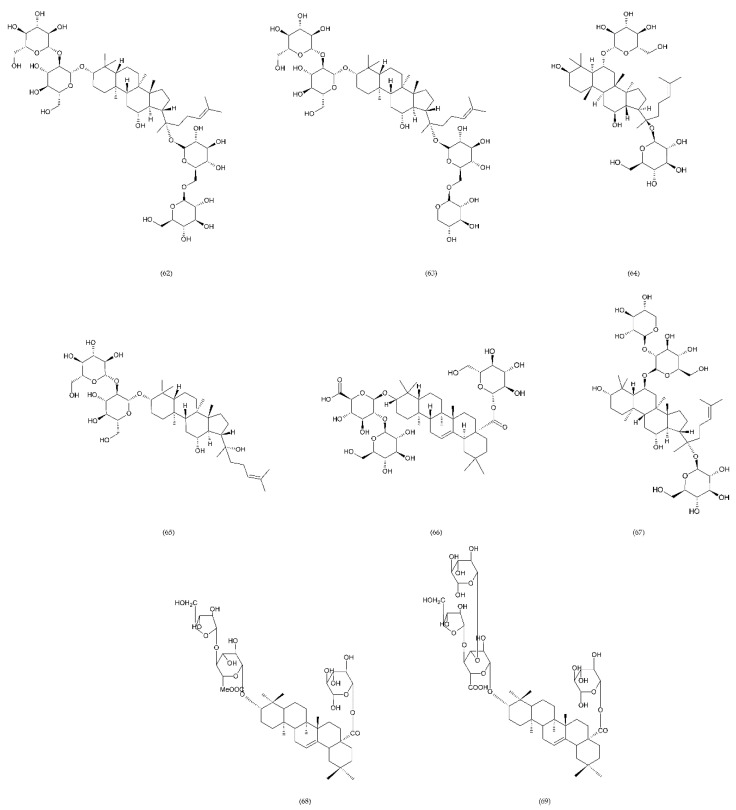
Chemical structure of saponins: ginsenoside rb1 (62), ginsenoside rb3 (63), ginsenoside rg1 (64), ginsenoside rg3 (65), ginsenoside Ro (66), notoginsenoside R1 (67), Panax bipinnatifidus, araloside A methyl ester (68), and Panax bipinnatifidus stipuleanoside R2 (69).

Saponins *Panax notoginseng* (PNS) are part of a traditional herb used in Chinese medicine to treat blood clotting, swelling, and pain relief. The main components of *Panax notoginseng* are ginsenoside Rg1 (64), ginsenoside Rb1 (62), and notoginsenoside R1 (67) (Figure 19). *Panax* saponins inhibit platelet aggregation, preventing thrombosis, but also regulate many signalling pathways by acting on cardiovascular protection. In addition, they show anti-inflammatory effects mediated by peroxisome proliferator-activated receptor γ (PPAR-γ) in human endothelial cells. In vitro inhibited platelet aggregation induced by thrombin overexpression of PPAR-γ increased expression of PI3Kβ/Akt/eNOS pathway in platelets. In vivo studied saponin *Panax notoginseng* reversed thrombin-induced hypercoagulable state saponins *Panax* induced PPAR-γ activation and also had a key role in the PI3Kβ/Akt/eNOS pathway [286]. Other PNS saponins have anti-inflammatory, antioxidant action, they inhibit platelet aggregation, regulate glucose and blood pressure, inhibit neuronal apoptosis, and provide neuronal protection [287].

The platelet aggregation inhibiting properties have been demonstrated by the next two saponins (Figure 19) isolated from *Panax bipinnatifidus*, a plant used in traditional Vietnamese medicine. These include araloside A methyl ester (68) and stipuleanoside R2 (69), which are the main components isolated from this plant [288]. However, their mechanism of action has not yet been described. In turn, ginsenoside Ro (66), *Panax ginseng* oleanane-type saponin, inhibiting IIb/IIIa mediated fibrinogen binding by preventing platelet aggregation may be an effective anticoagulant [285].

#### 7.2.2. *Liriope muscari*

**Figure 20:**
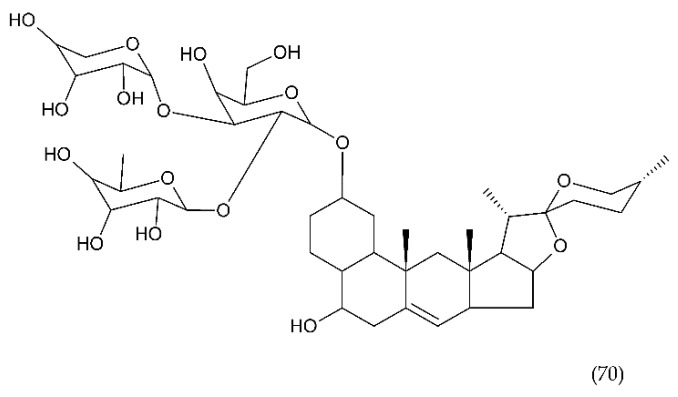
Chemical structure of saponin D39 (70).

Anticoagulant properties were demonstrated by natural saponin D39 (70) (Figure 20) isolated from *Liriope muscari,* and the mechanism of its action is associated with the non-muscle myosin heavy chain IIa (NMMHC IIA). In turn, NMMHC IIA is important in the expression of TF and, consequently, venous thrombosis. Using HUVEC cells has been shown that saponin D39 inhibits TF expression and has anticoagulant activity. In addition, saponin D39 inhibited thrombus formation by affecting the Akt/GSK3β and NF-κB signalling pathways by NMMHC IIA. It was suggested that D39 could be a potential therapy for DVT and other cardiovascular diseases [289].

The steroid saponin DT-13 isolated from *Liriope muscari* has been used in traditional Chinese medicine to treat a variety of diseases such as pharyngitis, bronchitis, pneumonia, cough, and cardiovascular diseases. DT-13 also shows anticoagulant properties as demonstrated in vivo in the classical model of IVC ligation thrombosis in mice and rats. DT-13 inhibits venous thrombosis partly by reducing gene expressions of TF and decreasing IL-6 expression [290].

#### 7.2.3. Steroidal Sapogenin

**Figure 21:**
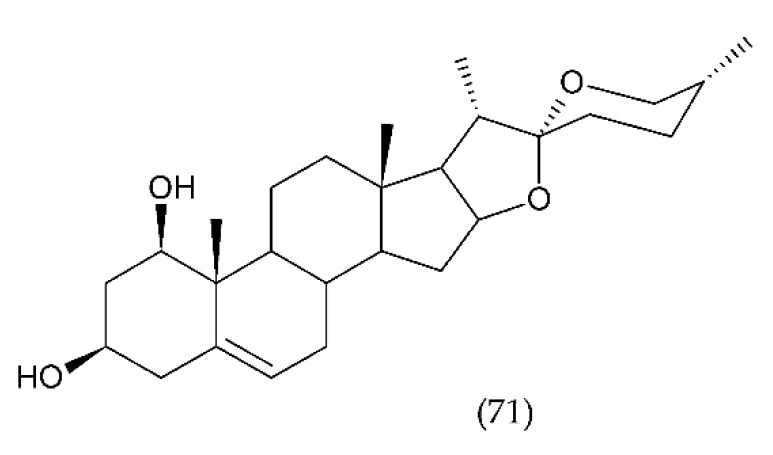
Chemical structure of ruscogenin (RUS) (71).

Ruscogenin (RUS) (71) (Figure 21), a steroidal sapogenin isolated from *Ruscus aculeatus,* is also the main component of the traditional Chinese herb Radix *Ophiopogon japonicus*, showing significant anti-inflammatory and anti-coagulant effects [291,292]. In addition, ruscogenin significantly inhibits leukocyte adhesion to the human umbilical vein endothelial line (ECV304). It has been used for thousands of years in the treatment of acute and chronic inflammatory and cardiovascular diseases [293].

Diosgenin and other steroidal saponins isolated from this plant are demonstrated to have anti-thrombotic activity. From few isolated saponins, especially Diosgenyl saponin, which has the linkage of the sugar chains at the C-3 position of diosgenin, had anti-thrombotic activity and significantly inhibited platelet aggregation in vitro and in vivo. This saponin did not act via the extrinsic coagulation pathway but rather by inhibiting molecular targets in intrinsic coagulation pathways. Diosgenyl saponin protected mice against pulmonary thromboembolism induced by collagen and epinephrine. It demonstrates anti-thrombotic activity through inhibiting FVIII activities and platelet aggregation [294].

#### 7.2.4. Other Saponins

*Polygala fallax* Hesml root saponin extract (PTS) significantly prolonged coagulation time in mice, reduce thrombus length and inhibited carrageenan-induced inflammation without affecting thrombus formation. PTS had anticoagulant and anticoagulant effects, probably via the endogenous coagulation system [295]. Seeds of chestnut (*Aesculus hippocastanum* L., *Sapindaceae*) contain approx. 3–5% saponins and flavonoids: quercetin and derivatives of kaempferol, proanthocyanidins, and sterols. They are used to isolate escin, a saponin used to treat chronic venous insufficiency. Escin has a positive effect on venous tone, rheological properties, and blood clotting. In addition, it has anti-inflammatory and anti-oedema properties [296]. This chapter describes the different classes of saponins found in plants and their antithrombotic properties.

### 7.3. Stevioside and Derivertes

**Figure 22:**
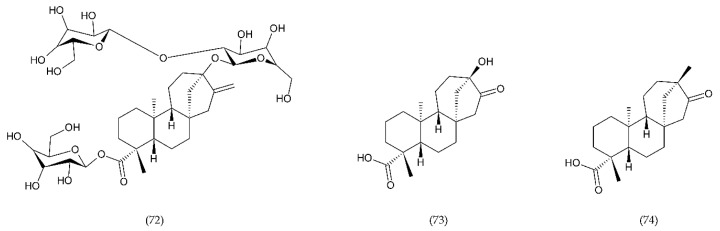
Chemical structure of diterpenoids: stevioside (72), steviol (73), and isosteviol (74).

Plant-derived diterpenoids (Figure 22) demonstrate a wide range of biological activity and exhibit antitumor, antibacterial, antihyperglycemic, anti-inflammatory and immunomodulatory, antifungal, antiparasitic, antiviral, antiallergic, antispasmodic, antihyperglycemic, and other effects [297]. Stevioside (72) is diterpenoid found in significant amounts in the leaves of the herb Stevia (Stevia rebaudiana Bertoni) [298], which is native to the region of South America. Stevia belongs to the Asteraceae chrysanthemum family. Stevioside is a sweet non-toxic and non-mutagenic drug used in patients with metabolic problems in type II diabetes, phenylketonuria, and obesity [299]. Stevioside (Figure 22) reduces the influx of calcium, lowers blood pressure, increases the insulin signal, increases the antioxidant potential in the aorta, and inhibits the process of atherosclerosis. The antioxidant properties and the reduction of oxidative damage were previously well documented in many studies [300,301]. Recently, the mechanism of its action has been explained, which is associated with the enhancement of the endogenous related pathway with nuclear factor erythroid 2-related factor 2 (Nrf2), which is one of the key transcription factors in the human body. Nrf2 also regulates the expression of enzymes to counteract oxidative stress [302].

In contrast, isosteviol (74) caused an increase in vasodilatation, a decrease in angiotensin II, and cell proliferation, as well as a decrease in the concentration of reactive oxygen and endothelin-1. A decrease in extracellular signal-regulated kinase (ERK), hyperplasia, and hypertrophy were also observed in vascular smooth muscle cells [303]. Stevioside has been shown to inhibit the expression of IL-6, IL-1β and TNF-α and LPS-induced initiation, NF-κB, inhibitor of kappa B (IκBα) degradation, c-Jun N-terminal kinase (JNK), p38 and ERK phosphorylation in a dose-dependent manner. In addition, it was shown that the anti-inflammatory activity can be exerted by inhibiting NF-κB activation and MAPK and expression of pro-inflammatory cytokines [304]. Stevioside inhibited the release of proinflammatory cytokines and inhibited LPS-induced COX-2 and iNOS expression in mice in vivo [305].

Steviol (73), similar to stevioside, significantly inhibited TNF-α induced IL-8 expression in human colon cancer cells, Caco-2, T84 10 and HT29 [155]. Steviol’s immunomodulatory activity was associated with NF-κB signalling [306]. In turn, Boonkaewwan et al. [304] conducting studies on the colon carcinoma Caco-2 cell line showed that stevioside and steviol inhibited LPS-induced release of IL-6, TNF-α and IL-1β by affecting the expression of the cytokine gene through the signal pathway IκBα/NF-κB.

Isosteviol (ent-16-oxobeyran-19-acid) is a tetracyclic skeletal diterpenoid of the Beyerene type, obtained by acid hydrolysis of stevioside [307]. Isosteviol has biological activity, including anti-inflammatory, antihypertensive, controlling blood lipids, immunoregulation, antiviral, antibacterial, and others [308]. It has been reported that steviol significantly inhibited proliferation of cancer cells by induction of apoptosis. In addition, steviol leads to the generation of ROS in cancer cells. Isosteviol also showed strong antithrombotic properties by selectively inhibiting FXa against a serine protease panel and anticoagulant activity by significantly prolonging PT and aPTT time as well as inhibiting ADP-induced platelet aggregation [309].

## 8. Conclusions

This paper reviews the pathophysiology of clot formation involving blood cells and cell components, critical factors released from activated cells and damaged cells as well as all already known drugs and methods to prevent or cure thromboembolism in all stages.

A new insight into the physiology of platelets and their involvement in inflammatory response and activation of clotting has been presented. These two processes have an important role in defending the host from infection. Their role in innate immunity in recognising pathogens, signal transduction, and cytokine/chemokine release is essential in thrombus formation, preventing the spread of microorganisms. In addition to platelets, other blood morphotic components such as leukocytes, erythrocytes, and vascular endothelial cells, along with activation of the coagulation system, have a significant role in the development of blood clots.

We considered the specific and key role of ECs in signalling mechanisms in which cyclophilin A (CypA) is secreted and ROS are generated. Both factors are sufficient to initiate vasculitis, secret MMPs, and induce changes in the structure of the vessel wall. The studies presented thus far show the role of platelets in the stages of inflammation, which indicates that they can be the target of an appropriate group of drugs. Undoubtedly, antiplatelet agents are effective in the treatment of thrombosis, but the problem is an increased risk of bleeding. However, a more accurate understanding of the processes associated with thrombosis and haemostasis has allowed the adoption of new strategies that are tested on animal models. Those are antiplatelet agents that inhibit thrombosis but do not affect haemostasis. In addition to antiplatelet drugs, there are also new strategies targeting clotting factors. An example is FXI, the inhibition of which may prove to be safer than the use of FXa inhibitors or thrombin. The FXI factor is important for the stabilisation and growth of the thrombus, but less important for haemostasis.

Even though modern medicine has a wide range of anticoagulant drugs, thrombosis still leads to cardiovascular diseases, myocardial infarction, and stroke. While significant advances have been made in understanding the mechanisms of thrombus formation and the pathophysiology of thrombosis, current prevention or treatment drugs that have been used for many years have been replaced by newer ones that show greater potential for the prevention and treatment of thrombosis with modest but gradual improvement. High hopes are associated with compounds of natural origin, which show a strong anticoagulant effect both at the experimental and clinical stage and can be used in the prophylactic or supportive methods of treatment of thrombotic diseases.

## Figures and Tables

**Table 1:** Effect of drug anticoagulants for atherothrombotic diseases.

Anticoagulants				
Drug	Formulation	Mechanisms of Action	Route of Administration	References
Apixaban BMS-562247	10 mg twice daily for 1 week, then 5 mg twice daily 2.5 mg twice daily for 2–5 weeks	FXa inhibitor	oral anticoagulant	[155,178,179,180]
Edoxaban DU-176	15, 30, and 60 mg daily	FXa inhibitor	oral anticoagulant	[178,179,181,182]
Rivaroxaban BAY59-7939	20 mg daily 15 mg BD for 3 weeks, then 20 mg 10 mg daily for 2–5 weeks	FXa inhibitor	oral anticoagulant	[155,178,179,180]
Low molecular weight heparin (LMWH) Anti-Xa	Dosing of LMWH depends on the drug used: Dalteparin: 200 U/kg OD or 100 U/kg BID Enoxaparin: 1.5 mg/kg OD or 1 mg/kg BID Tinzaparin: 175 U/kg OD Nadroparin: 171 U/kg OD	Indirect FXa inhibitor	parenteral formulation	[155,183]
Fondaparinux	- Under 50 kg: 5 mg once a day - 50 to 100 kg: 7.5 mg once a day - Over 100 kg: 10 mg once a day	Indirect FXa inhibitor	parenteral formulation	[155,169,184]
Idraparinux SR34006	2.5 mg once weekly	Indirect FXa inhibitor	parenteral formulation	[169]
Darexaban YM-150	30 mg or 60 mg daily	FXa inhibitor	oral anticoagulant	[155,185]
Betrixaban	15 mg twice daily or 40 mg twice daily	FXa inhibitor	oral anticoagulant	[155]
Nokxaban GC-2107 GCC-4401C	20 mg and 40 mg administered in a fasted state is comparable to rivaroxaban 10 mg and 20 mg in a fed state 2.5–80 mg single dose	FXa inhibitor	oral anticoagulant	[155,186,187,188]
Hirudin	1.75 mg/kg/h	Thrombin inhibitor	parenteral formulation	[169]
Bivalirudin	0.75 mg/kg intravenous bolus dose followed immediately 1.75 mg/kg/h intravenous infusion for the duration of the procedure	Thrombin inhibitor	parenteral formulation	[169]
Argatroban	2 µg/kg/min administered as a constant infusion	Thrombin inhibitor	parenteral formulation	[169]
Unfractionated heparin UFH	80 units/kg	Indirect FXa inhibitor, thrombin inhibitor	parenteral formulation	[169,189]
Dabigatran BIBR 1048 BIBR 953	150 mg twice daily (after 5 days of parenteral anticoagulation) 110 mg loading dose then 220 mg daily for 9–34 days	FIIa, thrombin inhibitor	oral anticoagulant	[155,169,178,179,190]
Warfarin	5–10 mg daily	FIIa, FVIIa, FIXa, FXa inhibitor	oral anticoagulant	[155,178]

**Table 2:** Antiplatelets drugs in the treatment of atherothrombosis, the doses used, and the receptor inhibitors/antagonists.

Antiplatelet Drugs				
Drug	Formulation	Mechanisms of Action	Route of Administration	References
Abciximab	Bolus 0.25 mg/kg injection then 0.125 µg/kg/min infusion	Glycoprotein IIb/IIIa inhibitor	parenteral formulation	[202,209]
Eptifibatide	180 µg/kg before the start of percutaneous coronary intervention followed by a continuous infusion of 2 µg/kg/min with another 180 µg/kg IV bolus given 10 min	Glycoprotein IIb/IIIa inhibitor	parenteral formulation	[202,210]
Tirofiban	0.15 µg/kg/min for 24–36 h	Glycoprotein IIb/IIIa inhibitor	parenteral formulation	[202,211]
Clopidogrel	300 mg–600 mg loading dose followed by 75 mg daily, in conjunction with aspirin, ideally for up to 12 months	P2Y12 receptor antagonist	oral anticoagulant	[202,212]
Prasugrel LY-640315	60 mg loading dose 10 mg once-daily maintenance dose	P2Y12 receptor antagonist	oral anticoagulant	[202,213]
Cangrelor	Bolus 30 µg/kg injection then 4 µg/kg/min infusion	P2Y12 receptor blockade	parenteral formulation	[202,214]
Acetylsalicylic Acid ASA	In Europe loading dose 300 mg, maintenance dose 75 mg	Irreversible acetylation and inhibition of COX enzyme	oral anticoagulant	[202,213]
Ticagrelor	180 mg loading dose 90 mg twice-daily maintenance dose	P2Y12 receptor blockade	oral anticoagulant	[202,213]
Cilostazol	100 mg twice daily	PDE inhibitor	oral anticoagulant	[215]
Dipyridamole	ASA 25 mg and dipyridamole 200 mg 225 mg with 1000 mg ASA	PDE inhibitor	oral anticoagulant	[216]
Ticlopidine	250 mg twice daily for 4 weeks 300 mg daily	ADP receptor inhibitor	oral anticoagulant	[217]
Selatogrel ACT-246475	8 mg or 16 mg single dose	P2Y12 receptor antagonist	oral anticoagulant	[218,219]

## Abbreviations

AA	Arachidonic acid
ACE	Angiotensin-converting enzyme
ADP	Adenosine diphosphate
AGEs	Advanced glycation end products
Akt	Protein kinase B
AMI	Acute myocardial infarction
aPTT	Activated partial thromboplastin time
apoAI	Apoplipoprotein A-I
APS	Antiphospholipid syndrome
ASA	Acetylsalicylic acid
ASO	Antisense oligonucleotides
AT	Antithrombin
ATP	Adenosine triphosphate
cAMP	Cyclic adenosine monophosphate
CAT	Catalase
CCL	Chemokine (C-C motif) ligand
CD	Cluster of differentiation
CDT	Catheter-directed thrombolysis
CEP	Centrosome-associated protein
CHCL1	Chemokine induces the infiltration of neutrophils
CLEC	C-type lectin-like receptor
COX	Cyclooxygenase
CT	Computed tomography
CX3CL1	Fractalkine
CX3CR1	CX3C chemokine receptor 1
CXCL	Chemokine CXC ligand-1
CYP	Cytochrome P450
CypA	Cyclophilin A
DNA	Deoxyribonucleic acid
DVT	Deep vein thrombosis
eATP	Extracellular ATP
ECE	Endothelin converting enzyme
ECs	Endothelial cells
ED	Endothelial dysfunction
EDCF	Endothelium-derived contracting factor
EDGF	Endothelium-derived growth factor
EDHF	Endothelium-derived hyperpolarizing factor
EGF	Epidermal growth factor
EMP	Microparticles of endothelial cells
EMT	Epithelial-mesenchymal transition
eNOS	Endothelial nitric oxide synthase
ERK	Extracellular signal-regulated kinase
ETC	Electron transport chain
FGF	Fibroblast growth factor
GP	Glycoprotein
GSK	Glycogen synthase kinase
H_2_O	Water
H_2_O_2_	Hydrogen peroxide
Hb	Haemoglobin
HClO	Hypochlorous acid
HIF	Hypoxia inducible factor
HNE	4-hydroxy-2-nonenal
HO^•^	Hydroxyl radical
HO_2_^•^	Hydroperoxyl radical
Hp	Haptoglobin
HRE	Hormone response element
HsCRP	High-sensitivity C-reactive protein
5HT2A	Serotonin receptor 2A
HUVEC	Human umbilical vein endothelial cells
Hx	Hemopexin
IAV	Influenza A virus
ICAM	Intercellular adhesion molecule
iCypA	Extracellular cyclophilin A
IDUs	Intravenous drug users
IFN	Interferon
IGF	Insulin-like growth factor
IL	Interleukin
iNOS	Induced nitric oxide synthase
INR	International normalized ratio
IVC	Inferior vena cava
IκBα	Inhibitor of kappa B
JAK-2	Janus kinase 2
JNK	C-Jun N-terminal kinase
LMWH	Low molecular weight heparin
LOX	Lipoxygenase
LPS	Lipopolysaccharide
MAPK	Mitogen-activated protein kinases
MCP	Monocyte chemoattractant protein
metHb	Methaemoglobin
MHC	Major histocompatibility complex
MIDAS	Metal ion-dependent adhesion site
MIP-2	Macrophage inflammatory protein 2
MKP-1	Mitogen-activated protein kinases phosphatase 1
MMP	Matrix metalloproteinase
MPO	Myeloperoxidase
MPV	Mean platelets volume
mPTP	Mitochondrial permeability transition pore
MRI	Magnetic resonance imaging
NAD^+^	Nicotinamide-adenine dinucleotide phosphate (oxidised form)
NADPH	Nicotinamide-adenine dinucleotide phosphate (reduced form)
NAPc2	Nematode anticoagulant protein c2
NETs	Neutrophil extracellular traps
NF-κB	Nuclear factor kappa B
NLRP3	Inflammasome is component of the innate immune system
NMMHC IIA	Non-muscle myosin heavy chain IIa
NO^•^	Nitric oxide
NOX	NADPH oxidase
Nrf2	Nuclear factor erythroid 2-related factor 2
NSCLC	Non-small-cell lung carcinoma
O_2_	Oxygen
^1^O_2_	Singlet oxygen
O_2_^•^^−^	Superoxide anion radical
ONOO^−^	Peroxynitrite
ox-L	Oxidised lipids
ox-P	Oxidised proteins
oxyHb	Oxyhaemoglobin
PAF	Platelet activating factor
PAI-I	Plasminogen activator inhibitor-1
PAR	Protease-activated receptor
PAT	Percutaneous aspiration thrombectomy
PCDT	Catheter-directed pharmacomechanical thrombolysis
PCI	Percutaneous coronary intervention
PD-L1	Programmed death-ligand 1
PDE3	Phosphodiesterase type 3
PDGF	Platelet-derived growth factor
PDI	Protein disulfide-isomerase
PE	Pulmonary embolism
PF	Platelet factor
PGE	Prostaglandin E
PGG	Prostaglandin G
PGH	Prostaglandin H
PGI	Prostacycline
PI3Kβ	Phosphatidylinositol 3 kinase β
PKA	Protein kinase A
PKC	Protein kinase C
PLa	Activated platelets
PLA	Phospholipase A
PLC	Phospholipase C
PLCγ2	Phospholipase Cγ2
PMN	Polymorphonuclear leukocyte
PMQ	Pentamethyl quercetin
PNS	Saponins *Panax notoginseng*
PPAR-γ	Peroxisome proliferator-activated receptor γ
PSGL-1	P-selectin glycoprotein ligand-1
PT	Prothrombin time
PTS	*Polygala fallax* Hesml root saponin extract
RBCs	Red blood cells
RNA	Ribonucleic acid
ROS	Reactive oxygen species
SBSI	Simultaneous decrease in prostacyclin synthase
SLO	Streptolysin O
SLS	Streptolysin S
SOD	Superoxide dismutase
STAT3	Signal transducer and activator of transcription 3
STEC	Shigatoxigenic *Escherichia coli*
TF	Tissue factor
TGF-β	Transforming growth factor beta
TLR	Toll-like receptor
TNF-α	Tumour necrosis factor alfa
t-PA	Tissue plasminogen activator
TRAP	Thrombin receptor activator peptide
TTP	Thrombocytopenic purpura
TXA2	Tromboxan A2
UFH	Unfractionated heparin
UV	Ultraviolet
VCAM	Vascular cell adhesion molecule
VEGF	Vascular endothelial growth factor
VKORC1	Vitamin K epoxide reductase complex
VSMCs	Vascular smooth muscle cells
VTE	Venous thromboembolism
vWF	Von Willebrand factor

## References

[1] Weitz J.I., Chan N.C. (2019). Advances in Antithrombotic Therapy. Arterioscler. Thromb. Vasc. Biol..

[2] Essien E.-O., Rali P., Mathai S.C. (2019). Pulmonary Embolism. Med Clin. N. Am..

[3] Flumignan C.D., Flumignan R.L., Baptista-Silva J.C. (2016). Antiplatelet agents for the treatment of deep venous thrombosis. Cochrane Database Syst. Rev..

[4] Wattanakit K., Lutsey P.L., Bell E.J., Gornik H., Cushman M., Heckbert S.R., Rosamond W.D., Folsom A.R. (2012). Association between cardiovascular disease risk factors and occurrence of venous thromboembolism. Thromb. Haemost..

[5] Stone J., Hangge P., Albadawi H., Wallace A., Shamoun F., Knuttien M.G., Naidu S., Oklu R. (2017). Deep vein thrombosis: Pathogenesis, diagnosis, and medical management. Cardiovasc. Diagn. Ther..

[6] Criqui M.H., Denenberg J.O., Bergan J., Langer R.D., Fronek A. (2007). Risk factors for chronic venous disease: The San Diego Population Study. J. Vasc. Surg..

[7] Kwiatkowska W., Knysz B., Gąsiorowski J., Witkiewicz W. (2015). Deep vein thrombosis of the lower limbs in intravenous drug users. Postępy Hig. Med. Doświadczalnej.

[8] Pearson J.D. (1994). 1 Endothelial cell function and thrombosis. Baillière’s Clin. Haematol..

[9] Van Hinsbergh V.W.M. (2012). Endothelium—Role in regulation of coagulation and inflammation. Semin. Immunopathol..

[10] Wolberg A.S., Campbell R.A. (2008). Thrombin generation, fibrin clot formation and hemostasis. Transfus. Apher. Sci..

[11] Weisel J.W., Litvinov R.I. (2017). Fibrin Formation, Structure and Properties. Subcell Biochem..

[12] Petrillo G., Cirillo P., Luana D’Ascoli G.-, Maresca F., Ziviello F., Chiariello M. (2010). Tissue Factor/Factor FVII Complex Inhibitors in Cardiovascular Disease. Are Things Going Well?. Curr. Cardiol. Rev..

[13] Budnik I., Brill A. (2018). Immune Factors in Deep Vein Thrombosis Initiation. Trends Immunol..

[14] Koupenova M., Kehrel B.E., Corkrey H.A., Freedman J.E. (2016). Thrombosis and platelets: An update. Eur. Heart J..

[15] Tanaka K.A., Key N.S., Levy J.H. (2009). Blood Coagulation: Hemostasis and Thrombin Regulation. Anesth. Analg..

[16] MCKenzie P.J. (1991). Deep venous thrombosis and anaesthesia. Br. J. Anaesth..

[17] Branchford B.R., Carpenter S.L. (2018). The Role of Inflammation in Venous Thromboembolism. Front. Pediatrics.

[18] Iba T., Levy J.H. (2018). Inflammation and thrombosis: Roles of neutrophils, platelets and endothelial cells and their interactions in thrombus formation during sepsis. J. Thromb. Haemost..

[19] Ivanov I.I., Apta B.H.R., Bonna A.M., Harper M.T. (2019). Platelet P-selectin triggers rapid surface exposure of tissue factor in monocytes. Sci. Rep..

[20] Waldron B., Moll S. (2014). A patient’s guide to recovery after deep vein thrombosis or pulmonary embolism. Circulation.

[21] Swystun L.L., Liaw P.C. (2016). The role of leukocytes in thrombosis. Blood.

[22] Brill A., Suidan G.L., Wagner D.D. (2013). Hypoxia, such as encountered at high altitude, promotes deep vein thrombosis in mice. J. Thromb. Haemost..

[23] Gupta N., Sahu A., Prabhakar A., Chatterjee T., Tyagi T., Kumari B., Khan N., Nair V., Bajaj N., Sharma M. (2017). Activation of NLRP3 inflammasome complex potentiates venous thrombosis in response to hypoxia. Proc. Natl. Acad. Sci. USA.

[24] Malone P.C. (2006). The aetiology of deep venous thrombosis. QJM.

[25] Esmon C.T. (2009). Basic mechanisms and pathogenesis of venous thrombosis. Blood Rev..

[26] Li C., Jackson R.M. (2002). Reactive species mechanisms of cellular hypoxia-reoxygenation injury. Am. J. Physiol. Cell Physiol..

[27] Pinsky D.J., Naka Y., Liao H., Oz M.C., Wagner D.D., Mayadas T.N., Johnson R.C., Hynes R.O., Heath M., Lawson C.A. (1996). Hypoxia-induced exocytosis of endothelial cell weibel-palade bodies: A mechanism for rapid neutrophil recruitment after cardiac preservation. J. Clin. Investig..

[28] Michiels C., Arnould T., Remacle J. (2000). Endothelial cell responses to hypoxia: Initiation of a cascade of cellular interactions. Biochim. Et Biophys. Acta BbaMol. Cell Res..

[29] Wang X., Liu K., Li B., Li Y., Ye K., Qi J., Wang Y. (2015). Macrophages Aggravate Hypoxia-Induced Cardiac Microvascular Endothelial Cell Injury via Peroxynitrite: Protection by Tongxinluo. Cell Commun. Adhes..

[30] Thompson L.F., Eltzschig H.K., Ibla J.C., Van De Wiele C.J., Resta R., Morote-Garcia J.C., Colgan S.P. (2004). Crucial Role for Ecto-5′-Nucleotidase (CD73) in Vascular Leakage during Hypoxia. J. Exp. Med..

[31] Eltzschig H.K., Abdulla P., Hoffman E., Hamilton K.E., Daniels D., Schönfeld C., Löffler M., Reyes G., Duszenko M., Karhausen J. (2005). HIF-1–dependent repression of equilibrative nucleoside transporter (ENT) in hypoxia. J. Exp. Med..

[32] Eckle T., Faigle M., Grenz A., Laucher S., Thompson L.F., Eltzschig H.K. (2008). A2B adenosine receptor dampens hypoxia-induced vascular leak. Blood.

[33] Blouin C.C., Pagé E.L., Soucy G.M., Richard D.E. (2004). Hypoxic gene activation by lipopolysaccharide in macrophages: Implication of hypoxia-inducible factor 1α. Blood.

[34] Kruger B., Krick S., Dhillon N., Lerner S.M., Ames S., Bromberg J.S., Lin M., Walsh L., Vella J., Fischereder M. (2009). Donor Toll-like receptor 4 contributes to ischemia and reperfusion injury following human kidney transplantation. Proc. Natl. Acad. Sci. USA.

[35] Baldea I., Teacoe I., Olteanu D.E., Vaida-Voievod C., Clichici A., Sirbu A., Filip G.A., Clichici S. (2018). Effects of different hypoxia degrees on endothelial cell cultures—Time course study. Mech. Ageing Dev..

[36] Vallabhapurapu S., Karin M. (2009). Regulation and Function of NF-κB Transcription Factors in the Immune System. Annu. Rev. Immunol..

[37] Incalza M.A., D’Oria R., Natalicchio A., Perrini S., Laviola L., Giorgino F. (2018). Oxidative stress and reactive oxygen species in endothelial dysfunction associated with cardiovascular and metabolic diseases. Vasc. Pharmacol..

[38] Mold C., Morris C.A. (2001). Complement activation by apoptotic endothelial cells following hypoxia/reoxygenation. Immunology.

[39] HART M. (2004). Initiation of complement activation following oxidative stress.In vitro and in vivo observations. Mol. Immunol..

[40] Poyton R.O., Ball K.A., Castello P.R. (2009). Mitochondrial generation of free radicals and hypoxic signaling. Trends Endocrinol. Metab..

[41] Castello P.R., David P.S., McClure T., Crook Z., Poyton R.O. (2006). Mitochondrial cytochrome oxidase produces nitric oxide under hypoxic conditions: Implications for oxygen sensing and hypoxic signaling in eukaryotes. Cell Metab..

[42] Palacios-Callender M., Hollis V., Mitchison M., Frakich N., Unitt D., Moncada S. (2007). Cytochrome c oxidase regulates endogenous nitric oxide availability in respiring cells: A possible explanation for hypoxic vasodilation. Proc. Natl. Acad. Sci. USA.

[43] Nishino T., Okamoto K., Eger B.T., Pai E.F., Nishino T. (2008). Mammalian xanthine oxidoreductase—Mechanism of transition from xanthine dehydrogenase to xanthine oxidase. FEBS J..

[44] Lundberg J.O., Weitzberg E., Gladwin M.T. (2008). The nitrate-nitrite-nitric oxide pathway in physiology and therapeutics. Nat. Rev. Drug Discov..

[45] Circu M.L., Aw T.Y. (2010). Reactive oxygen species, cellular redox systems, and apoptosis. Free Radic. Biol. Med..

[46] Spooner R., Yilmaz Ö. (2011). The Role of Reactive-Oxygen-Species in Microbial Persistence and Inflammation. Int. J. Mol. Sci..

[47] Ramachandran G. (2014). Gram-positive and gram-negative bacterial toxins in sepsis. Virulence.

[48] Hynes W., Sloan M., Ferretti J.J., Stevens D.L., Fischetti V.A. (2016). Secreted Extracellular Virulence Factors. Streptococcus Pyogenes: Basic Biology to Clinical Manifestations [Internet].

[49] Jürgens D. (2002). Production and characterization of Escherichia coli enterohemolysin and its effects on the structure of erythrocyte membranes. Cell Biol. Int..

[50] Krause M., Barth H., Schmidt H. (2018). Toxins of Locus of Enterocyte Effacement-Negative Shiga Toxin-Producing Escherichia coli. Toxins.

[51] Jiang N., Tan N.S., Ho B., Ding J.L. (2007). Respiratory protein–generated reactive oxygen species as an antimicrobial strategy. Nat. Immunol..

[52] Alayash A.I., Patel R.P., Cashon R.E. (2001). Redox Reactions of Hemoglobin and Myoglobin: Biological and Toxicological Implications. Antioxid. Redox Signal..

[53] Coates C.J., Decker H. (2017). Immunological properties of oxygen-transport proteins: Hemoglobin, hemocyanin and hemerythrin. Cell. Mol. Life Sci..

[54] Bahl N., Winarsih I., Tucker-Kellogg L., Ding J.L. (2014). Extracellular haemoglobin upregulates and binds to tissue factor on macrophages: Implications for coagulation and oxidative stress. Thromb. Haemost..

[55] Yang Y., Tang H. (2016). Aberrant coagulation causes a hyper-inflammatory response in severe influenza pneumonia. Cell. Mol. Immunol..

[56] Barnes M., Heywood A.E., Mahimbo A., Rahman B., Newall A.T., Macintyre C.R. (2015). Acute myocardial infarction and influenza: A meta-analysis of case–control studies. Heart.

[57] Rose J.J., Voora D., Cyr D.D., Lucas J.E., Zaas A.K., Woods C.W., Newby L.K., Kraus W.E., Ginsburg G.S. (2015). Gene Expression Profiles Link Respiratory Viral Infection, Platelet Response to Aspirin, and Acute Myocardial Infarction. PLoS ONE.

[58] Hauguel-Moreau M., El Hajjam M., De Baynast Q., Vieillard-Baron A., Lot A.-S., Chinet T., Mustafic H., Bégué C., Carlier R.Y., Geri G. (2020). Occurrence of pulmonary embolism related to COVID-19. J. Thromb. Thrombolysis.

[59] Koupenova M., Clancy L., Corkrey H.A., Freedman J.E. (2018). Circulating Platelets as Mediators of Immunity, Inflammation, and Thrombosis. Circ. Res..

[60] Zawilska K., Dmoszynska A., Robak T. (2015). Fizjologia hemostazy. Podstawy Hematologii.

[61] Korzonek-Szlacheta I., Zubelewicz-Szkodzińska B., Gąsior M. (2018). Płytki krwi—Ogniwo łączące zakrzepicę ze stanem zapalnym. Folia Cardiol..

[62] Violi F., Pignatelli P. (2012). Platelet Oxidative Stress and Thrombosis. Thromb. Res..

[63] Seno T., Inoue N., Gao D., Okuda M., Sumi Y., Matsui K., Yamada S., Hirata K., Kawashima S., Tawa R. (2001). Involvement of NADH/NADPH Oxidase in Human Platelet ROS Production. Thromb. Res..

[64] Qiao J., Arthur J.F., Gardiner E.E., Andrews R.K., Zeng L., Xu K. (2018). Regulation of platelet activation and thrombus formation by reactive oxygen species. Redox Biol..

[65] Stirban A., Gawlowski T., Roden M. (2014). Vascular effects of advanced glycation endproducts: Clinical effects and molecular mechanisms. Mol. Metab..

[66] Beristain-Covarrubias N., Perez-Toledo M., Thomas M.R., Henderson I.R., Watson S.P., Cunningham A.F. (2019). Understanding Infection-Induced Thrombosis: Lessons Learned From Animal Models. Front. Immunol..

[67] Guo Y., Nguyen K.-A., Potempa J. (2010). Dichotomy of gingipains action as virulence factors: From cleaving substrates with the precision of a surgeon’s knife to a meat chopper-like brutal degradation of proteins. Periodontology 2000.

[68] Proulx F., Seidman E.G., Karpman D. (2001). Pathogenesis of Shiga Toxin-Associated Hemolytic Uremic Syndrome. Pediatric Res..

[69] Goldsmith H.L., Turitto V.T. (1986). Rheological aspects of thrombosis and haemostasis: Basic principles and applications. ICTH-Report--Subcommittee on Rheology of the International Committee on Thrombosis and Haemostasis. Thromb. Haemost..

[70] Rana A., Westein E., Niego B., Hagemeyer C.E. (2019). Shear-Dependent Platelet Aggregation: Mechanisms and Therapeutic Opportunities. Front. Cardiovasc. Med..

[71] Ruggeri Z.M., Orje J.N., Habermann R., Federici A.B., Reininger A.J. (2006). Activation-independent platelet adhesion and aggregation under elevated shear stress. Blood.

[72] Spiel A.O., Gilbert J.C., Jilma B. (2008). Von Willebrand Factor in Cardiovascular Disease. Circulation.

[73] Gragnano F., Sperlongano S., Golia E., Natale F., Bianchi R., Crisci M., Fimiani F., Pariggiano I., Diana V., Carbone A. (2017). The Role of von Willebrand Factor in Vascular Inflammation: From Pathogenesis to Targeted Therapy. Mediat. Inflamm..

[74] Gorog D.A. (2003). Coronary angioplasty enhances platelet reactivity through von Willebrand factor release. Heart.

[75] Blair T.A., Moore S.F., Williams C.M., Poole A.W., Vanhaesebroeck B., Hers I. (2014). Phosphoinositide 3-Kinases p110α and p110β Have Differential Roles in Insulin-Like Growth Factor-1–Mediated Akt Phosphorylation and Platelet Priming. Arterioscler. Thromb. Vasc. Biol..

[76] Hathcock J.J. (2006). Flow Effects on Coagulation and Thrombosis. Arterioscler. Thromb. Vasc. Biol..

[77] Fogelson A.L., Neeves K.B. (2015). Fluid Mechanics of Blood Clot Formation. Annu. Rev. Fluid Mech..

[78] Lebas H., Yahiaoui K., Martos R., Boulaftali Y. (2019). Platelets Are at the Nexus of Vascular Diseases. Front. Cardiovasc. Med..

[79] Montoro-García S., Schindewolf M., Stanford S., Larsen O., Thiele T. (2016). The Role of Platelets in Venous Thromboembolism. Semin. Thromb. Hemost..

[80] Widlansky M.E., Gokce N., Keaney J.F., Vita J.A. (2003). The clinical implications of endothelial dysfunction. J. Am. Coll. Cardiol..

[81] Fan L.M., Douglas G., Bendall J.K., McNeill E., Crabtree M.J., Hale A.B., Mai A., Li J.-M., McAteer M.A., Schneider J.E. (2014). Endothelial Cell–Specific Reactive Oxygen Species Production Increases Susceptibility to Aortic Dissection. Circulation.

[82] Castro-Ferreira R., Cardoso R., Leite-Moreira A., Mansilha A. (2018). The Role of Endothelial Dysfunction and Inflammation in Chronic Venous Disease. J. Vasc. Surg. Venous Lymphat. Disord..

[83] Echeverría C., Montorfano I., Hermosilla T., Armisén R., Velásquez L.A., Cabello-Verrugio C., Varela D., Simon F. (2014). Endotoxin Induces Fibrosis in Vascular Endothelial Cells through a Mechanism Dependent on Transient Receptor Protein Melastatin 7 Activity. PLoS ONE.

[84] Deswal A., Petersen N.J., Feldman A.M., Young J.B., White B.G., Mann D.L. (2001). Cytokines and Cytokine Receptors in Advanced Heart Failure. Circulation.

[85] Cines D.B., Pollak E.S., Buck C.A., Loscalzo J., Zimmerman G.A., McEver R.P., Pober J.S., Wick T.M., Konkle B.A., Schwartz B.S. (1998). Endothelial cells in physiology and in the pathophysiology of vascular disorders. Blood.

[86] Fioranelli M., Bottaccioli A.G., Bottaccioli F., Bianchi M., Rovesti M., Roccia M.G. (2018). Stress and Inflammation in Coronary Artery Disease: A Review Psychoneuroendocrineimmunology-Based. Front. Immunol..

[87] Wheway J., Latham S.L., Combes V., Grau G.E.R. (2014). Endothelial Microparticles Interact with and Support the Proliferation of T Cells. J. Immunol..

[88] Allen R.C., Stephens J.T. (2011). Myeloperoxidase Selectively Binds and Selectively Kills Microbes. Infect. Immun..

[89] Case A. (2017). On the Origin of Superoxide Dismutase: An Evolutionary Perspective of Superoxide-Mediated Redox Signaling. Antioxidants.

[90] Brand M.D. (2016). Mitochondrial generation of superoxide and hydrogen peroxide as the source of mitochondrial redox signaling. Free Radic. Biol. Med..

[91] Phaniendra A., Jestadi D.B., Periyasamy L. (2015). Free Radicals: Properties, Sources, Targets, and Their Implication in Various Diseases. Indian J. Clin. Biochem..

[92] Bielski B.H.J., Cabelli D.E. (1995). Superoxide and Hydroxyl Radical Chemistry in Aqueous Solution. Active Oxygen in Chemistry.

[93] Li Y., Zhu H., Kuppusamy P., Zweier J., Trush M. (2016). Mitochondrial Electron Transport Chain-Derived Superoxide Exits Macrophages: Implications for Mononuclear Cell-Mediated Pathophysiological Processes. React. Oxyg. Species.

[94] Babior B.M. (2004). NADPH oxidase. Curr. Opin. Immunol..

[95] Bienert G.P., Schjoerring J.K., Jahn T.P. (2006). Membrane transport of hydrogen peroxide. Biochim. Biophys. Acta Bba Biomembr..

[96] Quinn M.T., Gauss K.A. (2004). Structure and regulation of the neutrophil respiratory burst oxidase: Comparison with nonphagocyte oxidases. J. Leukoc. Biol..

[97] Barrett C.D., Hsu A.T., Ellson C.D., Y. Miyazawa B., Kong Y.-W., Greenwood J.D., Dhara S., Neal M.D., Sperry J.L., Park M.S. (2018). Blood clotting and traumatic injury with shock mediates complement-dependent neutrophil priming for extracellular ROS, ROS-dependent organ injury and coagulopathy. Clin. Exp. Immunol..

[98] Nowak W.N., Deng J., Ruan X.Z., Xu Q. (2017). Reactive Oxygen Species Generation and Atherosclerosis. Arterioscler. Thromb. Vasc. Biol..

[99] Galley J.C., Straub A.C. (2017). Redox Control of Vascular Function. Arterioscler. Thromb. Vasc. Biol..

[100] Aldosari S., Awad M., Harrington E., Sellke F., Abid M. (2018). Subcellular Reactive Oxygen Species (ROS) in Cardiovascular Pathophysiology. Antioxidants.

[101] Schreml S., Szeimies R.M., Prantl L., Karrer S., Landthaler M., Babilas P. (2010). Oxygen in acute and chronic wound healing. Br. J. Dermatol..

[102] Arvieux J., Regnault V., Hachulla E., Darnige L., Berthou F., Youinou P. (2001). Oxidation of beta2-glycoprotein I (beta2GPI) by the hydroxyl radical alters phospholipid binding and modulates recognition by anti-beta2GPI autoantibodies. Arthritis Res. Ther..

[103] Turrens J.F. (2003). Mitochondrial formation of reactive oxygen species. J. Physiol..

[104] Lambeth J.D. (2007). Nox enzymes, ROS, and chronic disease: An example of antagonistic pleiotropy. Free Radic. Biol. Med..

[105] Lassègue B., Griendling K.K. (2010). NADPH Oxidases: Functions and Pathologies in the Vasculature. Arterioscler. Thromb. Vasc. Biol..

[106] Doughan A.K., Harrison D.G., Dikalov S.I. (2008). Molecular Mechanisms of Angiotensin II–Mediated Mitochondrial Dysfunction. Circ. Res..

[107] Chen F., Haigh S., Barman S., Fulton D.J.R. (2012). From form to function: The role of Nox4 in the cardiovascular system. Front. Physiol..

[108] Cervantes Gracia K., Llanas-Cornejo D., Husi H. (2017). CVD and Oxidative Stress. J. Clin. Med..

[109] Weyemi U., Dupuy C. (2012). The emerging role of ROS-generating NADPH oxidase NOX4 in DNA-damage responses. Mutat. Res. Rev. Mutat. Res..

[110] Kim Y.-M., Kim S.-J., Tatsunami R., Yamamura H., Fukai T., Ushio-Fukai M. (2017). ROS-induced ROS release orchestrated by Nox4, Nox2, and mitochondria in VEGF signaling and angiogenesis. Am. J. Physiol. Cell Physiol..

[111] Saini R., Singh S. (2019). Inducible nitric oxide synthase: An asset to neutrophils. J. Leukoc. Biol..

[112] Fuchs T.A., Brill A., Duerschmied D., Schatzberg D., Monestier M., Myers D.D., Wrobleski S.K., Wakefield T.W., Hartwig J.H., Wagner D.D. (2010). Extracellular DNA traps promote thrombosis. Proc. Natl. Acad. Sci. USA.

[113] Gupta A.K., Joshi M.B., Philippova M., Erne P., Hasler P., Hahn S., Resink T.J. (2010). Activated endothelial cells induce neutrophil extracellular traps and are susceptible to NETosis-mediated cell death. FEBS Lett..

[114] Dikshit M., Kumari R., Srimal R.C. (1993). Pulmonary thromboembolism-induced alterations in nitric oxide release from rat circulating neutrophils. J. Pharmacol. Exp. Ther..

[115] Mitsuke Y., Lee J.-D., Shimizu H., Uzui H., Iwasaki H., Ueda T. (2001). Nitric oxide synthase activity in peripheral polymorphonuclear leukocytes in patients with chronic congestive heart failure. Am. J. Cardiol..

[116] Nguyen G.T., Green E.R., Mecsas J. (2017). Neutrophils to the ROScue: Mechanisms of NADPH Oxidase Activation and Bacterial Resistance. Front. Cell. Infect. Microbiol..

[117] Kirchner T., Möller S., Klinger M., Solbach W., Laskay T., Behnen M. (2012). The Impact of Various Reactive Oxygen Species on the Formation of Neutrophil Extracellular Traps. Mediat. Inflamm..

[118] Paiva C.N., Bozza M.T. (2014). Are Reactive Oxygen Species Always Detrimental to Pathogens?. Antioxid. Redox Signal..

[119] El-Benna J., Hurtado-Nedelec M., Marzaioli V., Marie J.-C., Gougerot-Pocidalo M.-A., Dang P.M.-C. (2016). Priming of the neutrophil respiratory burst: Role in host defense and inflammation. Immunol. Rev..

[120] Sheshachalam A., Srivastava N., Mitchell T., Lacy P., Eitzen G. (2014). Granule Protein Processing and Regulated Secretion in Neutrophils. Front. Immunol..

[121] Lassègue B., Sorescu D., Szöcs K., Yin Q.Q., Akers M., Zhang Y., Grant S.L., Lambeth J.D., Griendling K.K. (2001). Novel gp91phox homologues in vascular smooth muscle cells: Nox1 mediates angiotensin II-induced superoxide formation and redox-sensitive signaling pathways. Circ. Res..

[122] San José G., Bidegain J., Robador P.A., Díez J., Fortuño A., Zalba G. (2009). Insulin-induced NADPH oxidase activation promotes proliferation and matrix metalloproteinase activation in monocytes/macrophages. Free Radic. Biol. Med..

[123] Xue C., Sowden M.P., Berk B.C. (2018). Extracellular and Intracellular Cyclophilin A, Native and Post-Translationally Modified, Show Diverse and Specific Pathological Roles in Diseases. Arterioscler. Thromb. Vasc. Biol..

[124] Zhang C. (2008). The role of inflammatory cytokines in endothelial dysfunction. Basic Res. Cardiol..

[125] Yu W., Hu X., Wang M. (2018). Pterostilbene inhibited advanced glycation end products (AGEs)-induced oxidative stress and inflammation by regulation of RAGE/MAPK/NF-κB in RAW264.7 cells. J. Funct. Foods.

[126] Duque G.A., Descoteaux A. (2014). Macrophage Cytokines: Involvement in Immunity and Infectious Diseases. Front. Immunol..

[127] Alvarez B., Radi R. (2003). Peroxynitrite reactivity with amino acids and proteins. Amino Acids.

[128] Elsayed N.M. (1994). Toxicity of nitrogen dioxide: An introduction. Toxicology.

[129] Persinger R.L., Poynter M.E., Ckless K., Janssen-Heininger Y.M.W. (2002). Molecular mechanisms of nitrogen dioxide induced epithelial injury in the lung. Mol. Cell. Biochem..

[130] Gray B., Carmichael A.J. (1992). Kinetics of superoxide scavenging by dismutase enzymes and manganese mimics determined by electron spin resonance. Biochem. J..

[131] Crow J.P., Beckman J.S. (1995). Reactions between Nitric Oxide, Superoxide, and Peroxynitrite: Footprints of Peroxynitrite in Vivo. Adv. Pharmacol..

[132] Nauser T., Koppenol W.H. (2002). The Rate Constant of the Reaction of Superoxide with Nitrogen Monoxide: Approaching the Diffusion Limit. J. Phys. Chem. A.

[133] Münzel T., Sinning C., Post F., Warnholtz A., Schulz E. (2008). Pathophysiology, diagnosis and prognostic implications of endothelial dysfunction. Ann. Med..

[134] Gow A.J., Luchsinger B.P., Pawloski J.R., Singel D.J., Stamler J.S. (1999). The oxyhemoglobin reaction of nitric oxide. Proc. Natl. Acad. Sci. USA.

[135] Auten R.L., Davis J.M. (2009). Oxygen Toxicity and Reactive Oxygen Species: The Devil Is in the Details. Pediatric Res..

[136] Brzeszczynska J., Gwozdzinski K. (2008). Nitric oxide induced oxidative changes in erythrocyte membrane components. Cell Biol. Int..

[137] Ansari S.A., Pendurthi U.R., Rao L.V.M. (2017). The lipid peroxidation product 4-hydroxy-2-nonenal induces tissue factor decryption via ROS generation and the thioredoxin system. Blood Adv..

[138] Gutmann C., Siow R., Gwozdz A.M., Saha P., Smith A. (2020). Reactive Oxygen Species in Venous Thrombosis. Int. J. Mol. Sci..

[139] Golino P., Ragni M., Cirillo P., Avvdimento V.E., Feliciello A., Esposito N., Scognamiglio A., Trimarco B., Iaccarino G., Condorelli M. (1996). Effects of tissue factor induced by oxygen free radicals on coronary flow during reperfusion. Nat. Med..

[140] Cadroy Y., Dupouy D., Boneu B., Plaisancié H. (2000). Polymorphonuclear Leukocytes Modulate Tissue Factor Production by Mononuclear Cells: Role of Reactive Oxygen Species. J. Immunol..

[141] Herkert O., Diebold I., Brandes R.P., Hess J., Busse R., Görlach A. (2002). NADPH Oxidase Mediates Tissue Factor—Dependent Surface Procoagulant Activity by Thrombin in Human Vascular Smooth Muscle Cells. Circulation.

[142] Mackman N. (2008). Triggers, targets and treatments for thrombosis. Nature.

[143] Lichota A., Gwozdzinski L., Gwozdzinski K. (2019). Therapeutic potential of natural compounds in inflammation and chronic venous insufficiency. Eur. J. Med. Chem..

[144] Kovacs M.J., Kahn S.R., Wells P.S., Anderson D.A., Chagnon I., Le Gal G., Solymoss S., Crowther M., Perrier A., Ramsay T. (2010). Patients with a first symptomatic unprovoked deep vein thrombosis are at higher risk of recurrent venous thromboembolism than patients with a first unprovoked pulmonary embolism. J. Thromb. Haemost..

[145] Goktay A.Y., Senturk C. (2017). Endovascular Treatment of Thrombosis and Embolism. Adv. Exp. Med. Biol..

[146] Shatila W., Almanfi A., Massumi M., Dougherty K.G., Parekh D.R., Strickman N.E. (2016). Endovascular Treatment of Superior Vena Cava Syndrome via Balloon-in-Balloon Catheter Technique with a Palmaz Stent. Tex. Heart Inst. J..

[147] Ringleb P.A. (2006). Thrombolytics, Anticoagulants, and Antiplatelet Agents. Stroke.

[148] Sen C.K., Roy S., Rodriguez E., Losee J.E., Neligan P.C. (2012). Wound healing. Plastic Surgery.

[149] Kearon C., Akl E.A., Ornelas J., Blaivas A., Jimenez D., Bounameaux H., Huisman M., King C.S., Morris T.A., Sood N. (2016). Antithrombotic Therapy for VTE Disease. Chest.

[150] Lee W., Lee H., Kim M.-A., Choi J., Kim K.-M., Hwang J.S., Na M., Bae J.-S. (2017). Evaluation of novel factor Xa inhibitors from Oxya chinensis sinuosa with anti-platelet aggregation activity. Sci. Rep..

[151] Fischer P.M. (2018). Design of Small-Molecule Active-Site Inhibitors of the S1A Family Proteases as Procoagulant and Anticoagulant Drugs. J. Med. Chem..

[152] Zacconi F.C. (2018). FXa Direct Synthetic Inhibitors. Anticoagulant Drugs.

[153] Byon W., Garonzik S., Boyd R.A., Frost C.E. (2019). Apixaban: A Clinical Pharmacokinetic and Pharmacodynamic Review. Clin. Pharmacokinet..

[154] Bratsos S. (2019). Pharmacokinetic Properties of Rivaroxaban in Healthy Human Subjects. Cureus.

[155] Rao P.S.S., Burkart T. (2017). Advances in oral anticoagulation therapy—What’s in the pipeline?. Blood Rev..

[156] Gómez-Outes A., Terleira-Fernández A.I., Calvo-Rojas G., Suárez-Gea M.L., Vargas-Castrillón E. (2013). Dabigatran, Rivaroxaban, or Apixaban versus Warfarin in Patients with Nonvalvular Atrial Fibrillation: A Systematic Review and Meta-Analysis of Subgroups. Thrombosis.

[157] Bushnell C., McCullough L.D., Awad I.A., Chireau M.V., Fedder W.N., Furie K.L., Howard V.J., Lichtman J.H., Lisabeth L.D., Piña I.L. (2014). Guidelines for the Prevention of Stroke in Women. Stroke.

[158] Yee J., Kaide C. (2019). Emergency Reversal of Anticoagulation. West. J. Emerg. Med..

[159] Jiang H., Meng J., Guo T., Zhao J., Wang Y., Wang J., Qiu Y., Ding H. (2019). Comparison of Apixaban and Low Molecular Weight Heparin in Preventing Deep Venous Thrombosis after Total Knee Arthroplasty in Older Adults. Yonsei Med. J..

[160] Skelley J.W., Thomason A.R., Nolen J.C. (2018). Betrixaban (Bevyxxa): A Direct-Acting Oral Anticoagulant Factor Xa Inhibitor. Pharm. Ther..

[161] Iwatsuki Y., Sato T., Moritani Y., Shigenaga T., Suzuki M., Kawasaki T., Funatsu T., Kaku S. (2011). Biochemical and pharmacological profile of darexaban, an oral direct factor Xa inhibitor. Eur. J. Pharmacol..

[162] Shiraga T., Yajima K., Suzuki K., Suzuki K., Hashimoto T., Iwatsubo T., Miyashita A., Usui T. (2012). Identification of UDP-Glucuronosyltransferases Responsible for the Glucuronidation of Darexaban, an Oral Factor Xa Inhibitor, in Human Liver and Intestine. Drug Metab. Dispos..

[163] Hirayama F., Koshio H., Ishihara T., Hachiya S., Sugasawa K., Koga Y., Seki N., Shiraki R., Shigenaga T., Iwatsuki Y. (2011). Discovery of N-[2-Hydroxy-6-(4-methoxybenzamido)phenyl]-4- (4-methyl-1,4-diazepan-1-yl)benzamide (Darexaban, YM150) as a Potent and Orally Available Factor Xa Inhibitor. J. Med. Chem..

[164] Clayville L.R., Vogel Anderson K., Miller S.A., Onge E.L.S. (2011). New Options in Anticoagulation for the Prevention Of Venous Thromboembolism and Stroke. PT.

[165] Shirley M., Dhillon S. (2015). Edoxaban: A Review in Deep Vein Thrombosis and Pulmonary Embolism. Drugs.

[166] Brown D.G., Wilkerson E.C., Love W.E. (2015). A review of traditional and novel oral anticoagulant and antiplatelet therapy for dermatologists and dermatologic surgeons. J. Am. Acad. Dermatol..

[167] Székely O., Miyazawa K., Lip G.Y.H. (2018). Current and emerging pharmacotherapy for ischemic stroke prevention in patients with atrial fibrillation. Expert Opin. Pharmacother..

[168] Feng W., Wu K., Liu Z., Kong G., Deng Z., Chen S., Wu Y., Chen M., Liu S., Wang H. (2015). Oral direct factor Xa inhibitor versus enoxaparin for thromboprophylaxis after hip or knee arthroplasty: Systemic review, traditional meta-analysis, dose–response meta-analysis and network meta-analysis. Thromb. Res..

[169] Bates S.M., Weitz J.I. (2005). New anticoagulants: Beyond heparin, low-molecular-weight heparin and warfarin. Br. J. Pharmacol..

[170] Kamikubo Y., Mendolicchio G.L., Zampolli A., Marchese P., Rothmeier A.S., Orje J.N., Gale A.J., Krishnaswamy S., Gruber A., Østergaard H. (2017). Selective factor VIII activation by the tissue factor–factor VIIa–factor Xa complex. Blood.

[171] Markwardt F. (2002). Hirudin As Alternative Anticoagulant- A Historical Review. Semin. Thromb. Hemost..

[172] Dong H., Ren J.-X., Wang J.-J., Ding L.-S., Zhao J.-J., Liu S.-Y., Gao H.-M. (2016). Chinese Medicinal Leech: Ethnopharmacology, Phytochemistry, and Pharmacological Activities. Evid. Based Complementary Altern. Med..

[173] Zhang Z., Li Z., Li J., Liu L. (2018). Effects of Natural Hirudin and Low Molecular Weight Heparin in Preventing Deep Venous Thrombosis in Aged Patients with Intertrochanteric Fracture. Sci. Rep..

[174] Buck M.L. (2015). Bivalirudin as an Alternative to Heparin for Anticoagulation in Infants and Children. J. Pediatric Pharmacol. Ther..

[175] Kimmelstiel C., Zhang P., Kapur N.K., Weintraub A., Krishnamurthy B., Castaneda V., Covic L., Kuliopulos A. (2011). Bivalirudin Is a Dual Inhibitor of Thrombin and Collagen-Dependent Platelet Activation in Patients Undergoing Percutaneous Coronary Intervention. Circ. Cardiovasc. Interv..

[176] Qureshi W., Ali Z., Amjad W., Alirhayim Z., Farooq H., Qadir S., Khalid F., Al-Mallah M.H. (2016). Venous Thromboembolism in Cancer: An Update of Treatment and Prevention in the Era of Newer Anticoagulants. Front. Cardiovasc. Med..

[177] Koch A.W., Schiering N., Melkko S., Ewert S., Salter J., Zhang Y., McCormack P., Yu J., Huang X., Chiu Y.-H. (2019). MAA868, a novel FXI antibody with a unique binding mode, shows durable effects on markers of anticoagulation in humans. Blood.

[178] Di Minno A., Frigerio B., Spadarella G., Ravani A., Sansaro D., Amato M., Kitzmiller J.P., Pepi M., Tremoli E., Baldassarre D. (2017). Old and new oral anticoagulants: Food, herbal medicines and drug interactions. Blood Rev..

[179] Samuelson B.T., Cuker A. (2017). Measurement and reversal of the direct oral anticoagulants. Blood Rev..

[180] Cesarman-Maus G., Ruiz-Argüelles G.J. (2017). News in the Indications of Direct Oral Anticoagulants According to the American College of Chest Physicians 2016 Guidelines. Curr. Drug Metab..

[181] Ansell J.E., Bakhru S.H., Laulicht B.E., Steiner S.S., Grosso M.A., Brown K., Dishy V., Lanz H.J., Mercuri M.F., Noveck R.J. (2017). Single-dose ciraparantag safely and completely reverses anticoagulant effects of edoxaban. Thromb. Haemost..

[182] Okumura K., Lip G.Y.H., Akao M., Tanizawa K., Fukuzawa M., Abe K., Akishita M., Yamashita T. (2017). Edoxaban for the management of elderly Japanese patients with atrial fibrillation ineligible for standard oral anticoagulant therapies: Rationale and design of the ELDERCARE-AF study. Am. Heart J..

[183] Thomson P., Brocklebank C., Semchuk W. (2009). Treatment Dosing of Low-Molecular-Weight Heparins and the Dose Cap Dilemma: Considerations for Patients in Canada. Can. J. Hosp. Pharm..

[184] Nadar S.K., Goyal D., Shantsila E., Banerjee P., Lip G.Y. (2009). Fondaparinux: An overview. Expert Rev. Cardiovasc. Ther..

[185] Eriksson B., Agnelli G., Gallus A., Lassen M., Prins M., Renfurm R., Kashiwa M., Turpie A. (2014). Darexaban (YM150) versus enoxaparin for the prevention of venous thromboembolism after total hip arthroplasty: A randomised phase IIb dose confirmation study (ONYX-3). Thromb. Haemost..

[186] Dimitropoulos G., Rahim S.M.Z., Moss A.S., Lip G.Y.H. (2018). New anticoagulants for venous thromboembolism and atrial fibrillation: What the future holds. Expert Opin. Investig. Drugs.

[187] Choi H., Choi S., Kim Y., Lim H. (2016). Population Pharmacokinetic and Pharmacodynamic Modeling Analysis of GCC-4401C, a Novel Direct Factor Xa Inhibitor, in Healthy Volunteers. CPT Pharmacomet. Syst. Pharmacol..

[188] Roffel A., van Marle S., Choi S., Lee H., Dueker S., Tiessen R. (2017). Metabolism and excretion of GCC-4401C, a factor Xa inhibitor, in humans. Int. J. Pharmacokinet..

[189] Smythe M.A., Priziola J., Dobesh P.P., Wirth D., Cuker A., Wittkowsky A.K. (2016). Guidance for the practical management of the heparin anticoagulants in the treatment of venous thromboembolism. J. Thromb. Thrombolysis.

[190] Lippi G., Gosselin R., Favaloro E.J. (2019). Current and Emerging Direct Oral Anticoagulants: State-of-the-Art. Semin. Thromb. Hemost..

[191] Capodanno D., Ferreiro J.L., Angiolillo D.J. (2013). Antiplatelet therapy: New pharmacological agents and changing paradigms. J. Thromb. Haemost..

[192] McFadyen J.D., Schaff M., Peter K. (2018). Current and future antiplatelet therapies: Emphasis on preserving haemostasis. Nat. Rev. Cardiol..

[193] Harter K., Levine M., Henderson S. (2015). Anticoagulation Drug Therapy: A Review. West. J. Emerg. Med..

[194] Rubboli A., Patti G. (2018). What is the Role for Glycoprotein IIB/IIIA Inhibitor Use in the Catheterization Laboratory in the Current Era?. Curr. Vasc. Pharmacol..

[195] Eisert W.G. (2012). Dipyridamole in antithrombotic treatment. Adv. Interv. Cardiol..

[196] Kim H.-H., Liao J.K. (2008). Translational Therapeutics of Dipyridamole. Arterioscler. Thromb. Vasc. Biol..

[197] Goto S. (2005). Cilostazol: Potential mechanism of action for antithrombotic effects accompanied by a low rate of bleeding. Atheroscler. Suppl..

[198] Niu P.-P., Guo Z.-N., Jin H., Xing Y.-Q., Yang Y. (2016). Antiplatelet regimens in the long-term secondary prevention of transient ischaemic attack and ischaemic stroke: An updated network meta-analysis. BMJ Open.

[199] Steinhubl S.R., Badimon J.J., Bhatt D.L., Herbert J.-M., Lüscher T.F. (2007). Clinical evidence for anti-inflammatory effects of antiplatelet therapy in patients with atherothrombotic disease. Vasc. Med..

[200] Quinn M.J., Fitzgerald D.J. (1999). Ticlopidine and Clopidogrel. Circulation.

[201] Jiang M., You J.H. (2015). Review of pharmacoeconomic evaluation of genotype-guided antiplatelet therapy. Expert Opin. Pharmacother..

[202] Majithia A., Bhatt D.L. (2019). Novel Antiplatelet Therapies for Atherothrombotic Diseases. Arterioscler. Thromb. Vasc. Biol..

[203] Tscharre M., Michelson A.D., Gremmel T. (2020). Novel Antiplatelet Agents in Cardiovascular Disease. J. Cardiovasc. Pharmacol. Ther..

[204] Wong P., Crain E., Watson C., Yang W., Wexler R., Lam P., Rehfuss R., Schumacher W. (2013). Differential effects of P2Y1 versus P2Y12 receptor antagonism on thrombosis and bleeding in rabbits. Eur. Heart J..

[205] Yang W., Wang Y., Lai A., Qiao J.X., Wang T.C., Hua J., Price L.A., Shen H., Chen X., Wong P. (2014). Discovery of 4-Aryl-7-Hydroxyindoline-Based P2Y 1 Antagonists as Novel Antiplatelet Agents. J. Med. Chem..

[206] Kolandaivelu K., Bhatt D.L. (2019). Novel Antiplatelet Therapies. Platelets.

[207] Bach P., Antonsson T., Bylund R., Björkman J.-A., Österlund K., Giordanetto F., van Giezen J.J.J., Andersen S.M., Zachrisson H., Zetterberg F. (2013). Lead Optimization of Ethyl 6-Aminonicotinate Acyl Sulfonamides as Antagonists of the P2Y 12 Receptor. Separation of the Antithrombotic Effect and Bleeding for Candidate Drug AZD1283. J. Med. Chem..

[208] Hashemzadeh M., Furukawa M., Goldsberry S., Movahed M.R. (2008). Chemical structures and mode of action of intravenous glycoprotein IIb/IIIa receptor blockers: A review. Exp. Clin. Cardiol..

[209] Stoffer K., Shah S. (2019). Abciximab.

[210] Bansal A.B., Sattar Y., Jamil R.T. (1999). Eptifibatide. Drugs.

[211] Zhao C., Cheng G., He R., Guo H., Li Y., Lu X., Zhang Y., Qiu C. (2015). Effects of different routes of tirofiban injection on the left ventricular function and prognosis of patients with myocardial infarction treated with percutaneous coronary intervention. Exp. Ther. Med..

[212] Beavers C.J., Naqvi I.A. (1997). Clopidogrel. Drugs.

[213] Hermanides R.S., Kilic S., van ’t Hof A.W.J. (2018). Optimal pharmacological therapy in ST-elevation myocardial infarction—a review. Neth. Heart J..

[214] Layne K., Ferro A. (2017). Antiplatelet Therapy in Acute Coronary Syndrome. Eur. Cardiol. Rev..

[215] Balinski A.M., Preuss C.V. (1999). Cilostazol. Drugs Aging.

[216] Royston D. (2019). Anticoagulant and Antiplatelet Therapy. Pharmacology and Physiology for Anesthesia.

[217] Desager J.-P. (1994). Clinical Pharmacokinetics of Ticlopidine. Clin. Pharmacokinet..

[218] Juif P., Boehler M., Dobrow M., Ufer M., Dingemanse J. (2019). Clinical Pharmacology of the Reversible and Potent P2Y 12 Receptor Antagonist ACT-246475 After Single Subcutaneous Administration in Healthy Male Subjects. J. Clin. Pharmacol..

[219] Storey R.F., Gurbel P.A., ten Berg J., Bernaud C., Dangas G.D., Frenoux J.-M., Gorog D.A., Hmissi A., Kunadian V., James S.K. (2020). Pharmacodynamics, pharmacokinetics, and safety of single-dose subcutaneous administration of selatogrel, a novel P2Y12 receptor antagonist, in patients with chronic coronary syndromes. Eur. Heart J..

[220] Zhu J., Choi W.-S., McCoy J.G., Negri A., Zhu J., Naini S., Li J., Shen M., Huang W., Bougie D. (2012). Structure-Guided Design of a High-Affinity Platelet Integrin IIb 3 Receptor Antagonist That Disrupts Mg^2+^ Binding to the MIDAS. Sci. Transl. Med..

[221] Li J., Vootukuri S., Shang Y., Negri A., Jiang J., Nedelman M., Diacovo T.G., Filizola M., Thomas C.J., Coller B.S. (2014). RUC-4. Arterioscler. Thromb. Vasc. Biol..

[222] Vootukuri S., Li J., Nedelman M., Thomas C., Jiang J.-K., Babayeva M., Coller B.S. (2019). Preclinical studies of RUC-4, a novel platelet αIIbβ3 antagonist, in non-human primates and with human platelets. J. Clin. Transl. Sci..

[223] Heuberger D.M., Schuepbach R.A. (2019). Protease-activated receptors (PARs): Mechanisms of action and potential therapeutic modulators in PAR-driven inflammatory diseases. Thromb. J..

[224] Gryka R.J., Buckley L.F., Anderson S.M. (2017). Vorapaxar: The Current Role and Future Directions of a Novel Protease-Activated Receptor Antagonist for Risk Reduction in Atherosclerotic Disease. Drugs Rd.

[225] Wilson S.J., Ismat F.A., Wang Z., Cerra M., Narayan H., Raftis J., Gray T.J., Connell S., Garonzik S., Ma X. (2018). PAR4 (Protease-Activated Receptor 4) Antagonism With BMS-986120 Inhibits Human Ex Vivo Thrombus Formation. Arterioscler. Thromb. Vasc. Biol..

[226] Dumas M., Nadal-Wollbold F., Gaussem P., Perez M., Mirault T., Létienne R., Bourbon T., Grelac F., Le Grand B., Bachelot-Loza C. (2012). Antiplatelet and antithrombotic effect of F 16618, a new thrombin proteinase-activated receptor-1 (PAR1) antagonist. Br. J. Pharmacol..

[227] Chieng-Yane P., Bocquet A., Létienne R., Bourbon T., Sablayrolles S., Perez M., Hatem S.N., Lompré A.-M., Le Grand B., David-Dufilho M. (2011). Protease-Activated Receptor-1 Antagonist F 16618 Reduces Arterial Restenosis by Down-Regulation of Tumor Necrosis Factor α and Matrix Metalloproteinase 7 Expression, Migration, and Proliferation of Vascular Smooth Muscle Cells. J. Pharmacol. Exp. Ther..

[228] Di Serio C., Pellerito S., Duarte M., Massi D., Naldini A., Cirino G., Prudovsky I., Santucci M., Geppetti P., Marchionni N. (2007). Protease-Activated Receptor 1-Selective Antagonist SCH79797 Inhibits Cell Proliferation and Induces Apoptosis by a Protease-Activated Receptor 1-Independent Mechanism. Basic Clin. Pharmacol. Toxicol..

[229] Hosokawa K., Ohnishi T., Miura N., Sameshima H., Koide T., Tanaka K.A., Maruyama I. (2014). Antithrombotic effects of PAR1 and PAR4 antagonists evaluated under flow and static conditions. Thromb. Res..

[230] Chen Z., Li T., Kareem K., Tran D., Griffith B.P., Wu Z.J. (2019). The role of PI3K/Akt signaling pathway in non-physiological shear stress-induced platelet activation. Artif. Organs.

[231] Sturgeon S.A., Jones C., Angus J.A., Wright C.E. (2008). Advantages of a selective β-isoform phosphoinositide 3-kinase antagonist, an anti-thrombotic agent devoid of other cardiovascular actions in the rat. Eur. J. Pharmacol..

[232] Laurent P.-A., Séverin S., Hechler B., Vanhaesebroeck B., Payrastre B., Gratacap M.-P. (2015). Platelet PI3Kβ and GSK3 regulate thrombus stability at a high shear rate. Blood.

[233] Durrant T.N., Hers I. (2020). PI3K inhibitors in thrombosis and cardiovascular disease. Clin. Transl. Med..

[234] Nylander S., Kull B., Björkman J.A., Ulvinge J.C., Oakes N., Emanuelsson B.M., Andersson M., Skärby T., Inghardt T., Fjellström O. (2012). Human target validation of phosphoinositide 3-kinase (PI3K)β: Effects on platelets and insulin sensitivity, using AZD6482 a novel PI3Kβ inhibitor. J. Thromb. Haemost..

[235] Xu P.-F., Yang J.-A., Liu J.-H., Yang X., Liao J.-M., Yuan F.-E., Liu B.-H., Chen Q.-X. (2019). PI3Kβ inhibitor AZD6482 exerts antiproliferative activity and induces apoptosis in human glioblastoma cells. Oncol. Rep..

[236] Luci D., Jameson J.B., Yasgar A., Diaz G., Joshi N., Kantz A., Markham K., Perry S., Kuhn N., Yeung J. (2010). Discovery of ML355, a Potent and Selective Inhibitor of Human 12-Lipoxygenase.

[237] Adili R., Tourdot B.E., Mast K., Yeung J., Freedman J.C., Green A., Luci D.K., Jadhav A., Simeonov A., Maloney D.J. (2017). First Selective 12-LOX Inhibitor, ML355, Impairs Thrombus Formation and Vessel Occlusion In Vivo With Minimal Effects on Hemostasis. Arterioscler. Thromb. Vasc. Biol..

[238] Gilbert J.C., DeFeo-Fraulini T., Hutabarat R.M., Horvath C.J., Merlino P.G., Marsh H.N., Healy J.M., BouFakhreddine S., Holohan T.V., Schaub R.G. (2007). First-in-Human Evaluation of Anti–von Willebrand Factor Therapeutic Aptamer ARC1779 in Healthy Volunteers. Circulation.

[239] Huang R.-H., Fremont D.H., Diener J.L., Schaub R.G., Sadler J.E. (2009). A Structural Explanation for the Antithrombotic Activity of ARC1172, a DNA Aptamer that Binds von Willebrand Factor Domain A1. Structure.

[240] Spiel A.O., Mayr F.B., Ladani N., Wagner P.G., Schaub R.G., Gilbert J.C., Jilma B. (2009). The aptamer ARC1779 is a potent and specific inhibitor of von willebrand factor mediated ex vivo platelet function in acute myocardial infarction. Platelets.

[241] Jilma-Stohlawetz P., Gilbert J., Gorczyca M., Knöbl P., Jilma B. (2011). A dose ranging phase I/II trial of the von Willebrand factor inhibiting aptamer ARC1779 in patients with congenital thrombotic thrombo—Cytopenic purpura. Thromb. Haemost..

[242] Kageyama S., Yamamoto H., Nagano M., Arisaka H., Kayahara T., Yoshimoto R. (1997). Anti-thrombotic effects and bleeding risk of AJvW-2, a monoclonal antibody against human von Willebrand factor. Br. J. Pharmacol..

[243] Siller-Matula J.M., Merhi Y., Tanguay J.-F., Duerschmied D., Wagner D.D., McGinness K.E., Pendergrast P.S., Chung J.-K., Tian X., Schaub R.G. (2012). ARC15105 Is a Potent Antagonist of Von Willebrand Factor Mediated Platelet Activation and Adhesion. Arterioscler. Thromb. Vasc. Biol..

[244] Hanlon A., Metjian A. (2020). Caplacizumab in adult patients with acquired thrombotic thrombocytopenic purpura. Ther. Adv. Hematol..

[245] Bartunek J., Barbato E., Heyndrickx G., Vanderheyden M., Wijns W., Holz J.-B. (2013). Novel Antiplatelet Agents: ALX-0081, a Nanobody Directed towards von Willebrand Factor. J. Cardiovasc. Transl. Res..

[246] Zheng L., Mao Y., Abdelgawwad M.S., Kocher N.K., Li M., Dai X., Li B., Zheng X.L. (2016). Therapeutic efficacy of the platelet glycoprotein Ib antagonist anfibatide in murine models of thrombotic thrombocytopenic purpura. Blood Adv..

[247] Kong Y., Huo J., Xu W., Xiong J., Li Y., Wu W. (2009). A novel anti-platelet aggregation tripeptide from Agkistrodon acutus venom: Isolation and characterization. Toxicon.

[248] Lei X., Reheman A., Hou Y., Zhou H., Wang Y., Marshall A., Liang C., Dai X., Li B.X., Vanhoorelbeke K. (2014). Anfibatide, a novel GPIb complex antagonist, inhibits platelet adhesion and thrombus formation in vitro and in vivo in murine models of thrombosis. Thromb. Haemost..

[249] Li T.-T., Fan M.-L., Hou S.-X., Li X.-Y., Barry D.M., Jin H., Luo S.-Y., Kong F., Lau L.-F., Dai X.-R. (2015). A novel snake venom-derived GPIb antagonist, anfibatide, protects mice from acute experimental ischaemic stroke and reperfusion injury. Br. J. Pharmacol..

[250] Voors-Pette C., Lebozec K., Dogterom P., Jullien L., Billiald P., Ferlan P., Renaud L., Favre-Bulle O., Avenard G., Machacek M. (2019). Safety and Tolerability, Pharmacokinetics, and Pharmacodynamics of ACT017, an Antiplatelet GPVI (Glycoprotein VI) Fab. Arterioscler. Thromb. Vasc. Biol..

[251] Imaizumi T., Yoshida H., Satoh K. (2004). Regulation of CX3CL1/Fractalkine Expression in Endothelial Cells. J. Atheroscler. Thromb..

[252] Stolla M., Pelisek J., von Brühl M.-L., Schäfer A., Barocke V., Heider P., Lorenz M., Tirniceriu A., Steinhart A., Bauersachs J. (2012). Fractalkine Is Expressed in Early and Advanced Atherosclerotic Lesions and Supports Monocyte Recruitment via CX3CR1. PLoS ONE.

[253] Riopel M., Vassallo M., Ehinger E., Pattison J., Bowden K., Winkels H., Wilson M., de Jong R., Patel S., Balakrishna D. (2019). CX3CL1-Fc treatment prevents atherosclerosis in Ldlr KO mice. Mol. Metab..

[254] Gailani D., Gruber A. (2016). Factor XI as a Therapeutic Target. Arterioscler. Thromb. Vasc. Biol..

[255] Wallisch M., Lorentz C.U., Lakshmanan H.H.S., Johnson J., Carris M.R., Puy C., Gailani D., Hinds M.T., McCarty O.J.T., Gruber A. (2020). Antibody inhibition of contact factor XII reduces platelet deposition in a model of extracorporeal membrane oxygenator perfusion in nonhuman primates. Res. Pract. Thromb. Haemost..

[256] Büller H.R., Bethune C., Bhanot S., Gailani D., Monia B.P., Raskob G.E., Segers A., Verhamme P., Weitz J.I. (2015). Factor XI Antisense Oligonucleotide for Prevention of Venous Thrombosis. N. Engl. J. Med..

[257] Thomas D., Thelen K., Kraff S., Schwers S., Schiffer S., Unger S., Yassen A., Boxnick S. (2019). BAY 1213790, a fully human IgG1 antibody targeting coagulation factor XIa: First evaluation of safety, pharmacodynamics, and pharmacokinetics. Res. Pract. Thromb. Haemost..

[258] Woodruff R.S., Ivanov I., Verhamme I.M., Sun M.-F., Gailani D., Sullenger B.A. (2017). Generation and characterization of aptamers targeting factor XIa. Thromb. Res..

[259] Hayward N.J., Goldberg D.I., Morrel E.M., Friden P.M., Bokesch P.M. (2017). Phase 1a/1b study of EP-7041: A novel, potent, selective, small molecule FXIa inhibitor. Circulation.

[260] Phang M., Lazarus S., Wood L., Garg M. (2011). Diet and Thrombosis Risk: Nutrients for Prevention of Thrombotic Disease. Semin. Thromb. Hemost..

[261] Vazhappilly C.G., Ansari S.A., Al-Jaleeli R., Al-Azawi A.M., Ramadan W.S., Menon V., Hodeify R., Siddiqui S.S., Merheb M., Matar R. (2019). Role of flavonoids in thrombotic, cardiovascular, and inflammatory diseases. Inflammopharmacology.

[262] Freedman J.E. (2008). Oxidative Stress and Platelets. Arterioscler. Thromb. Vasc. Biol..

[263] Faggio C., Sureda A., Morabito S., Sanches-Silva A., Mocan A., Nabavi S.F., Nabavi S.M. (2017). Flavonoids and platelet aggregation: A brief review. Eur. J. Pharmacol..

[264] Maiti S., Nazmeen A., Medda N., Patra R., Ghosh T.K. (2019). Flavonoids green tea against oxidant stress and inflammation with related human diseases. Clin. Nutr. Exp..

[265] Kosakowska O. (2017). Experimental Paper. Intrapopulation variability of flavonoid content in roots of Baikal skullcap (Scutellaria baicalensis Georgi). Herba Pol..

[266] Ryu R., Jung U.J., Seo Y.-R., Kim H.-J., Moon B.S., Bae J.-S., Lee D.G., Choi M.-S. (2015). Beneficial effect of persimmon leaves and bioactive compounds on thrombosis. Food Sci. Biotechnol..

[267] Khan M.M., Quoc Tran B., Jang Y.-J., Park S.-H., Fondrie W.E., Chowdhury K., Hwan Yoon S., Goodlett D.R., Chae S.-W., Chae H.-J. (2017). Assessment of the Therapeutic Potential of Persimmon Leaf Extract on Prediabetic Subjects. Mol. Cells.

[268] Zhang Y.-X., Yang T.-T., Xia L., Zhang W.-F., Wang J.-F., Wu Y.-P. (2017). Inhibitory Effect of Propolis on Platelet Aggregation In Vitro. J. Healthc. Eng..

[269] Lu W.-J., Lin K.-C., Liu C.-P., Lin C.-Y., Wu H.-C., Chou D.-S., Geraldine P., Huang S.-Y., Hsieh C.-Y., Sheu J.-R. (2016). Prevention of arterial thrombosis by nobiletin: In vitro and in vivo studies. J. Nutr. Biochem..

[270] Ku S.-K., Bae J.-S. (2014). Antithrombotic activities of wogonin and wogonoside via inhibiting platelet aggregation. Fitoterapia.

[271] Liang M.-L., Da X.-W., He A.-D., Yao G.-Q., Xie W., Liu G., Xiang J.-Z., Ming Z.-Y. (2015). Pentamethylquercetin (PMQ) reduces thrombus formation by inhibiting platelet function. Sci. Rep..

[272] Cheng C., Zhang H., Li Y., Zhou Y., Lu W., Yao L. (2017). The effect of Diosmin on the blood proteome in a rat model of venous thrombosis. Int. J. Biol. Macromol..

[273] Hsia C.-W., Wu M.-P., Velusamy M., Hsia C.-H., Chou D.-S., Tsai C.-L., Hsu C.-Y., Jayakumar T., Chung C.-L., Sheu J.-R. (2018). Novel Therapeutic Agent against Platelet Activation In Vitro and Arterial Thrombosis In Vivo by Morin Hydrate. Int. J. Mol. Sci..

[274] Lee M.H., Son Y.K., Han Y.N. (2002). Tissue factor inhibitory flavonoids from the fruits ofChaenomeles sinensis. Arch. Pharmacal Res..

[275] Karlíčková J., Říha M., Filipský T., Macáková K., Hrdina R., Mladěnka P. (2015). Antiplatelet Effects of Flavonoids Mediated by Inhibition of Arachidonic Acid Based Pathway. Planta Med..

[276] Dasgupta A. (2019). Antiinflammatory Herbal Supplements. Translational Inflammation.

[277] Liakopoulou C., Kazazis C., Vallianou N.G. (2019). Silimarin and Cancer. Anti-Cancer Agents Med. Chem..

[278] Bijak M., Dziedzic A., Saluk-Bijak J. (2018). Flavonolignans reduce the response of blood platelet to collagen. Int. J. Biol. Macromol..

[279] Bijak M., Szelenberger R., Dziedzic A., Saluk-Bijak J. (2018). Inhibitory Effect of Flavonolignans on the P2Y12 Pathway in Blood Platelets. Molecules.

[280] Kim J.H., Lee J., Kang S., Moon H., Chung K.H., Kim K.R. (2016). Antiplatelet and Antithrombotic Effects of the Extract of Lindera obtusiloba Leaves. Biomol. Ther..

[281] Shi W., Liu L., Li J., Qu L., Pang X., Yu H., Zhang Y., Wang T. (2017). Bioactive flavonoids from Flos Sophorae. J. Nat. Med..

[282] Chen C., Yang F.-Q., Zhang Q., Wang F.-Q., Hu Y.-J., Xia Z.-N. (2015). Natural Products for Antithrombosis. Evid. Based Complementary Altern. Med..

[283] Kregiel D., Berlowska J., Witonska I., Antolak H., Proestos C., Babic M., Babic L., Zhang B. (2017). Saponin-Based, Biological-Active Surfactants from Plants. Application and Characterization of Surfactants.

[284] Singh D., Chaudhuri P.K. (2018). Structural characteristics, bioavailability and cardioprotective potential of saponins. Integr. Med. Res..

[285] Shin J.-H., Kwon H.-W., Cho H.-J., Rhee M.H., Park H.-J. (2016). Vasodilator-stimulated phosphoprotein-phosphorylation by ginsenoside Ro inhibits fibrinogen binding to αIIb/β3 in thrombin-induced human platelets. J. Ginseng Res..

[286] Shen Q., Li J., Zhang C., Wang P., Mohammed A., Ni S., Tang Z. (2017). Panax notoginseng saponins reduce high-risk factors for thrombosis through peroxisome proliferator-activated receptor -γ pathway. Biomed. Pharmacother..

[287] Xie W., Meng X., Zhai Y., Zhou P., Ye T., Wang Z., Sun G., Sun X. (2018). Panax Notoginseng Saponins: A Review of Its Mechanisms of Antidepressant or Anxiolytic Effects and Network Analysis on Phytochemistry and Pharmacology. Molecules.

[288] Thom V., Tung N., Van Diep D., Thuy D., Hue N., Long D., Tung B., Huyen P., Ly Huong D. (2018). Antithrombotic activity and saponin composition of the roots of Panax bipinnatifidus Seem. growing in Vietnam. Pharmacogn. Res..

[289] Zhai K., Zheng J., Tang Y., Li F., Lv Y., Zhang Y., Gao Z., Qi J., Yu B., Kou J. (2017). The saponin D39 blocks dissociation of non-muscular myosin heavy chain IIA from TNF receptor 2, suppressing tissue factor expression and venous thrombosis. Br. J. Pharmacol..

[290] Yu B., Tian Y., Ma S., Lin B., Kou J. (2013). Anti-thrombotic activity of DT-13, a saponin isolated from the root tuber of Liriope muscari. Indian J. Pharmacol..

[291] Kou J., Yu B., Xu Q. (2005). Inhibitory effects of ethanol extract from Radix Ophiopogon japonicus on venous thrombosis linked with its endothelium-protective and anti-adhesive activities. Vasc. Pharmacol..

[292] Kou J., Tian Y., Tang Y., Yan J., Yu B. (2006). Antithrombotic Activities of Aqueous Extract from Radix Ophiopogon japonicus and Its Two Constituents. Biol. Pharm. Bull..

[293] Liu C., Huang Y. (2016). Chinese Herbal Medicine on Cardiovascular Diseases and the Mechanisms of Action. Front. Pharmacol..

[294] Zhang R., Huang B., Du D., Guo X., Xin G., Xing Z., Liang Y., Chen Y., Chen Q., He Y. (2013). Anti-thrombosis effect of diosgenyl saponins in vitro and in vivo. Steroids.

[295] Kou J.P., Li J.F., Yan J., Zhu D.N. (2003). Effect of total saponin from root of Polygala fallax Hesml. (PTS) on coagulation and thrombosis. J. China Pharm. Univ..

[296] Yoshikawa M., Matsuda H., Oleszek W., Marston A. (2000). Chemical and pharmacological studies on triterpene saponins, escins, from horse chestnut seeds. Saponins in Food, Feedstuffs and Medicinal Plants.

[297] Wang M., Li H., Xu F., Gao X., Li J., Xu S., Zhang D., Wu X., Xu J., Hua H. (2018). Diterpenoid lead stevioside and its hydrolysis products steviol and isosteviol: Biological activity and structural modification. Eur. J. Med. Chem..

[298] Geuns J.M. (2003). Stevioside. Phytochemistry.

[299] Carrera-Lanestosa A., Moguel-Ordóñez Y., Segura-Campos M. (2017). Stevia rebaudiana Bertoni: A Natural Alternative for Treating Diseases Associated with Metabolic Syndrome. J. Med. Food.

[300] Ruiz-Ruiz J.C., Moguel-Ordoñez Y.B., Segura-Campos M.R. (2017). Biological activity of Stevia rebaudiana Bertoni and their relationship to health. Crit. Rev. Food Sci. Nutr..

[301] Salehi B., López M.D., Martínez-López S., Victoriano M., Sharifi-Rad J., Martorell M., Rodrigues C.F., Martins N. (2019). Stevia rebaudiana Bertoni bioactive effects: From in vivo to clinical trials towards future therapeutic approaches. Phytother. Res..

[302] KKak S., Muriel P. (2017). Nrf2: A key regulator of redox signaling in liver diseases. Liver Pathophysiology.

[303] Wang Z., Zhao Y. (2018). Gut microbiota derived metabolites in cardiovascular health and disease. Protein Cell.

[304] Boonkaewwan C., Burodom A. (2013). Anti-inflammatory and immunomodulatory activities of stevioside and steviol on colonic epithelial cells. J. Sci. Food Agric..

[305] Yingkun N., Zhenyu W., Jing L., Xiuyun L., Huimin Y. (2013). Stevioside Protects LPS-Induced Acute Lung Injury in Mice. Inflammation.

[306] Boonkaewwan C., Ao M., Toskulkao C., Rao M.C. (2008). Specific Immunomodulatory and Secretory Activities of Stevioside and Steviol in Intestinal Cells. J. Agric. Food Chem..

[307] Bridel M., Lavieille R. (1931). The sweet principle in Kaa-he-e (Stevia rebaudiana. Bertoni). II. Hydrolysis of stevioside by enzymes. III. Steviol by enzymic hydrolysis and isosteviol by acid hydrolysis. Bull. Soc. Chim. Biol..

[308] Ullah A., Munir S., Mabkhot Y., Badshah S. (2019). Bioactivity Profile of the Diterpene Isosteviol and its Derivatives. Molecules.

[309] Chen P., Zhang D., Li M., Wu Q., Lam Y.P.Y., Guo Y., Chen C., Bai N., Malhotra S., Li W. (2019). Discovery of novel, potent, isosteviol-based antithrombotic agents. Eur. J. Med. Chem..

